# Development of a protein synthesis network at the sarco/endoplasmic reticulum in adult cardiac myocytes

**DOI:** 10.1113/JP288658

**Published:** 2026-08-11

**Authors:** Christoph Sandmann, Sören Brandenburg, Dennis Uhlenkamp, Bianca Meyer, Clara Sandmann, Nicole Herzog, Julia Groß, Charlotta Funaya, Hugo A. Katus, Stephan E. Lehnart, Christopher C. Glembotski, Mirko Völkers, Shirin Doroudgar

**Affiliations:** ^1^ Department of Cardiology, Angiology, and Pneumology Heidelberg University Hospital Heidelberg Germany; ^2^ DZHK (German Centre for Cardiovascular Research), Partner Site Heidelberg/Mannheim Heidelberg Germany; ^3^ Heart Research Center Göttingen, Department of Cardiology & Pneumology University Medical Center Göttingen Göttingen Germany; ^4^ DZHK (German Centre for Cardiovascular Research), Partner Site Göttingen Göttingen Germany; ^5^ Electron Microscopy Core Facility Heidelberg University Heidelberg Germany; ^6^ Department of Internal Medicine and Translational Cardiovascular Research Center University of Arizona College of Medicine – Phoenix Phoenix USA

**Keywords:** cardiac myocytes, endoplasmic reticulum, protein synthesis, sarcoplasmic reticulum, translation, ventricular myocyte

## Abstract

**Abstract:**

The endoplasmic reticulum (ER) is the primary site for the synthesis and folding of membrane and secretory proteins, which together comprise a large fraction of the total protein output in mammalian cells. Striated muscle cells contain a specialized membrane system, the sarcoplasmic reticulum (SR), which regulates calcium homeostasis and contraction. However, the biochemical and physiological relationship between the ER and SR, as well as the extent to which both compartments contribute to protein synthesis, remain incompletely understood. Quantification of ER‐ and SR‐associated proteins in isolated ventricular cardiac myocytes revealed that the relative abundance of ER/SR‐resident protein quality control components and ribosomes decreased during postnatal maturation, whereas SR‐associated Ca^2^^+^‐handling proteins increased. Immunocytochemistry revealed that the membrane compartment prominent in early postnatal stages exhibits predominantly ER characteristics and diminishes during postnatal development. In adult cardiac myocytes, the SR becomes the dominant membrane network throughout the cell, while ER markers remain enriched in the perinuclear region. Immunocytochemistry further indicated that the ER and SR perform overlapping yet distinct specialized functions, with excitation–contraction coupling localized to the SR, and initiation of secretion concentrated within the ER. In adult ventricular cardiac myocytes, ribosomes and mRNA localize adjacent to both the ER and SR, indicating their roles as direct sites of localized protein synthesis and homeostasis. These findings demonstrate that molecular differentiation and structural organization of the ER/SR during cardiac muscle development culminate in a specialized protein synthesis network within the sarco/endoplasmic reticulum of adult myocytes.

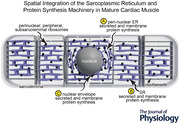

**Key points:**

- Although the sarcoplasmic reticulum (SR) is the established centre for calcium regulation in striated muscle, its role in membrane and secreted protein synthesis has remained unknown.
- It has remained unclear whether the endoplasmic reticulum (ER) and SR coexist as distinct membrane networks in cardiac myocytes or whether they form a single system that fulfills both calcium‐handling and protein synthesis functions.
- We found that postnatal cardiac maturation involves a major reorganization in which the centralized, ER‐dominant network of neonatal myocytes is replaced by an expansive, SR‐dominant network in the adult cell periphery, while ER markers become largely confined to the perinuclear region.
- Using stimulated emission depletion super resolution microscopy and electron microscopy, we demonstrated that active ribosomes and mRNA associate with the longitudinal SR but are spatially excluded from ryanodine receptor 2‐rich junctional zones.
- Our results establish that the SR functions as a specialized, distributed protein synthesis network that enables adult cardiac myocytes to maintain their highly organized cellular architecture through localized translation.

## Graphical abstract

**Abstract figure legend** Spatial integration of the sarcoplasmic reticulum and protein synthesis machinery in mature cardiac muscle: a schematic of the sarco/endoplasmic reticulum network in a cardiac myocyte, depicting the integrated relationships between the perinuclear endoplasmic reticulum (ER) (A), the sarcoplasmic reticulum (SR) (B), and the nuclear envelope (C). The localization of secreted and membrane protein synthesis, occurring across the nuclear envelope, perinuclear ER, and the SR, is depicted as a contiguous membranous system and is shaded purple.

## Introduction

The endoplasmic reticulum (ER) is a membrane network that when studded with ribosomes (known as the rough ER; rER), is the major site of secreted and membrane protein synthesis in many eukaryotic cell types (Porter & Kallman, 1952). Nevertheless, this essential organelle remains largely uncharacterized in cardiac muscle cells (myocytes). In striated muscle cells, including cardiac myocytes, a network of membranes called the sarcoplasmic reticulum (SR) has been defined and studied with important roles in muscle contraction (Porter & Palade, 1957). Biochemical and structural studies of developing muscle have shown that the development of the SR is the result of the specialization of the internal membrane system (Cusimano et al., 2009; Protasi et al., 1997; Schiaffino & Margreth, 1969; Takekura et al., 2001). Such a development has been proposed to arise from the differentiation of the ER, resulting in the formation of the developing SR continuous with the ER (Ezerman & Ishikawa, 1967; Schiaffino & Margreth, 1969; Wu & Bers, 2006). Therefore, the SR is often described as a ‘specialized form’ or ‘extension’ of the (smooth) ER of striated muscle cells (Greer et al., 2022; Rayavarapu et al., 2012; Rossi et al., 2022). However, the relative location and protein synthetic functions of the membrane network in cardiac myocytes remains unclear; specifically, whether both an ‘ER’ and a ‘SR’ network co‐exist in mature cardiac myocytes or whether a single highly specialized ‘SR’ membrane system fulfills both functions classically associated with the ER [e.g. (protein synthesis, secretion) and SR (Ca^2+^ homeostasis and excitation–contraction (EC) coupling)] remains unknown (Doroudgar & Glembotski, 2013; Michalak & Opas, 2009).

The mature SR makes contacts with invaginations of the plasma membrane, known as T‐tubules as well as sarcomeres, and is responsible for the storage, release, and reuptake of Ca^2+^. It is divided into two functionally and structurally distinct regions. The longitudinal SR contains few ryanodine receptors (RyR), which mediate calcium‐induced calcium release, but many SR Ca^2+^ pumps (i.e., the sarco/ER Ca^2+^‐ATPase; SERCA), which transport Ca^2+^ from the cytosol into the SR lumen. On the other hand, the junctional SR, which is situated in close apposition to T‐tubules, contains many RyR, for the fast, localized SR Ca^2+^ release during systole, resulting in fast activation of contraction (Inui et al., 1988; Somlyo, 1979). Although the function of the SR in cardiac contractile Ca^2+^‐handling is well established, its roles in protein synthesis, folding and quality control in cardiac myocytes are not as clear (Doroudgar & Glembotski, 2013).

In striated muscle, the majority of Ca^2+^‐handling proteins are localized within the SR lumen or membrane (Doroudgar & Glembotski, 2013; Rossi et al., 2022). This has led to the hypothesis that the SR may serve as a site for the localized synthesis of these proteins, similar to the co‐translational translocation of ER‐luminal and transmembrane proteins observed in the ER of non‐muscle cells. However, whether the SR in the mature cardiac myocyte functions as a direct site for protein synthesis remains a subject of ongoing investigation. In addition, because of its close interaction with the sarcomere, ribosomes attached to the SR may locally synthesize large sarcomeric proteins for structural homeostasis, as recently suggested for ribosomes and mRNA close to the sarcomere (Douvdevany et al., 2024; Lewis et al., 2018; Scarborough et al., 2021).

In the present study, we investigated the spatial distribution, postnatal maturation, and protein synthesis functions of the internal membrane network in primary rat cardiac myocytes. We found that, during cellular maturation, cardiac myocytes develop two distinct specialized membrane networks, with proteins traditionally associated with ER or SR functions localizing differentially. Importantly, we show that both the ER and SR membrane networks in mature adult cardiac myocytes are bound by ribosomes and actively engaged in mRNA translation.

## Methods

### Ethical approval

All animal experiments were approved by the local authorities (Regierungspräsidium Karlsruhe, Germany) and were performed in strict accordance with the Animal Research: Reporting of *in vivo* Experiments (ARRIVE) guidelines and Directive 2010/63/EU of the European Parliament. Terminal anaesthesia and protocols for killing rats were followed as described in the Methods to ensure rapid termination and the absence of pain or distress.

### Animal experiments

Experiments were performed using neonatal (1–3‐day‐old) and adult (8‐week‐old) Wistar rats (Janvier Labs, Le Genest‐Saint‐Isle, France) and adult (10‐week‐old) C57BL/6J or C57BL/6N mice. RiboTag mice (Rpl22^tm1.1Psam^/J) were used for cell‐type‐specific ribosome labelling. Animals were housed in a temperature‐controlled environment under a 12:12 h light/dark photocycle and *ad libitum* access to standard chow and water.

### Death and tissue collection

For neonatal tissue collection, rats were killed via decapitation, a method approved for this developmental stage to ensure rapid termination without chemical interference in protein synthesis networks. For adult animals, terminal anaesthesia was induced via intraperitoneal injection of sodium pentobarbital (200 mg kg^−1^; rats) or inhalation of isoflurane (5%; mice). A surgical plane of anaesthesia was confirmed by the absolute loss of the pedal withdrawal reflex (toe‐pinch). The method of death was defined as rapid thoracotomy and excision of the heart when under deep terminal anaesthesia. Death was confirmed by the permanent cessation of circulation and the absence of a visible heartbeat following cardiectomy.

### Cultured cardiac myocytes

Isolation of neonatal rat ventricular cardiac myocytes was performed as previously described (Hofmann, Serafin et al., 2024). Cells were isolated from one‐ to three‐day‐old Wistar rats (WISTAR RjHan:WI; Janvier Labs) via enzymatic digestion and purified by Percoll density gradient centrifugation. For western blotting experiments, cardiac myocytes were then plated at a density of 0.5 × 10^6^ cells per well on 34.8 mm plastic plates that had been pre‐treated with 5 µg mL^−1^ fibronectin (catalog no. F1141; Sigma‐Aldrich, St Louis, MO, USA) in serum‐free Dulbecco's modified Eagle's medium (DMEM)/F12 medium (catalog no. 11330032, Thermo Fisher Scientific, Waltham, MA, USA) for 1 h and then cultured in DMEM/F12 (1:1), containing 10% fetal bovine serum, 100 units mL^−1^ penicillin, 100 µg mL^−1^ streptomycin and 292 µg mL^−1^ glutamine (catalog no. 10378016; Thermo Fisher Scientific). Because the expression of several ER quality response proteins is regulated by medium serum levels, neonatal and adult cardiac myocytes were both cultured in serum‐free conditions. Twenty‐four hours after plating, neonatal cardiac myocytes were washed once with DMEM/F12 (1:1) and medium was changed to DMEM/F12 (1:1), containing 100 units mL^−1^ penicillin, 100 µg mL^−1^ streptomycin and 292 µg mL^−1^ glutamine for 24 h after which the cells were lysed for western blotting or fixed for immunocytofluorescence as described below. Adult mouse and rat ventricular myocytes were isolated from 10‐week‐old C57BL/6J mouse hearts or 8‐week‐old Wistar rats (WISTAR RjHan:WI; Janvier Labs), respectively, via enzymatic digestion as previously described (Jin et al., 2016; Völkers et al., 2011). Cells were plated in Medium 199 (catalog no. M7528; Sigma‐Aldrich) supplemented with 100 units mL^−1^ penicillin, 100 µg mL^−1^ streptomycin and 10 µg mL^−1^ laminin (catalog no. L2020; Sigma‐Aldrich). After 1 h, medium was changed to M199 medium supplemented with 100 units mL^−1^ penicillin and 100 µg mL^−1^ streptomycin and then lysed similar to neonatal cardiac myocytes.

### Immunocytofluorescence microscopy

Cardiac myocytes were isolated as described above. Cardiac myocytes were then plated on glass chamber slides that had been pre‐treated with fibronectin for 1 h. The cells were washed twice in phosphate‐buffered saline (PBS) and then fixed with 4% paraformaldehyde for 20 min. Slides were washed three times for 5–10 min in PBS and then incubated for 10 min in permeabilization buffer consisting of PBS, 0.2% Triton X‐100 and 0.1 m glycine. Then slides were washed once with PBS and then incubated with blocking buffer containing 10% horse serum and 0.2% Triton X‐100 in PBS for 1 h. Next, slides were incubated overnight at 4°C with primary antibody diluted in blocking buffer. Primary antibodies used were SERCA2 (catalog no. sc‐8095, N‐19; Santa Cruz Biotechnology, Santa Cruz, CA, USA; dilution 1:200), Ryanodine Receptor (catalog no. ab2827; Abcam, Cambridge, UK; dilution 1:50), KDEL (catalog no. 10C3, ADI‐SPA‐827; ENZO Life Sciences, Farmingdale, NY, USA; dilution 1:50), P‐S6 Ribosomal Protein^S235/236^ (catalog no. 2211; Cell Signaling Technology, Danvers, MA, USA), HA‐Tag (catalog no. C29F4, Cell Signaling Technology), GM130 (catalog no. 610822; BD Biosciences, San Jose, CA, USA; dilution 1:50), TGN38 (catalog no. sc‐33784, M‐290; Santa Cruz Biotechnology; dilution 1:50), CD63 (catalog no. MCA4754GA; Bio‐Rad, Hercules, CA, USA; dilution 1:50). On the next day, slides were washed three times for 5–10 min in PBS and then incubated with secondary antibodies in blocking buffer for 1 h. Secondary antibody used were fluorescein (FITC)‐conjugated AffiniPure Donkey Anti‐Mouse IgG (catalog no. 715‐095‐151; Jackson Immuno Research, West Grove, PA, USA; dilution 1:100), FITC‐conjugated AffiniPure Bovine Anti‐Goat IgG (catalog no. 808‐095‐180; Jackson Immuno Research; dilution 1:100), FITC‐conjugated AffiniPure Donkey Anti‐Rabbit IgG (catalog no. 711‐095‐152; Jackson Immuno Research; dilution 1:100), Cy3‐conjugated AffiniPure Donkey Anti‐Mouse IgG (catalog no. 715‐165‐151; Jackson Immuno Research; dilution 1:100), Cy3‐conjugated AffiniPure Donkey Anti‐Rabbit IgG (catalog no. 711‐165‐152; Jackson Immuno Research; dilution 1:100). The slides were then washed three times for 5–10 min in PBS and incubated in a 1:500 solution of 1 mM TO‐PRO‐3 iodide (catalog no. T3605; Thermo Fisher Scientific) diluted in PBS for 10 min. Then slides were washed twice in PBS for 10 min and covered with VECTASHIELD Antifade Mounting Medium (catalog no. H‐1000; Vector Laboratories, Newark, CA, USA) and a glass plate and visualized by microscopy. Poly(A) mRNA was visualized using a complementary RNA probe labelled with ATTO 647NN. Images were obtained using a DMi8 confocal laser scanning microscope (Leica, Wetzlar, Germany).

### Stimulated emission depletion (STED) microscopy

Ventricular cardiac myocytes from wild‐type mice (10‐week‐old, C57BL/6N background) were isolated using a Langendorff perfusion setup. Mouse hearts were cannulated and perfused with fresh, nominally Ca^2+^‐free isolation buffer (in mM: 120.4 NaCl, 14.7 KCl, 0.6 KH_2_PO_4_, 0.6 Na_2_HPO_4_, 1.2 MgSO_4_, 10 HEPES, 4.6 NaHCO_3_, 30 taurine, 10 2,3‐butanedione‐monoxime, 5.5 glucose, pH 7.4 with NaOH), followed by collagenase type II (Worthington Biochemical, Lakewood, NJ, USA) containing isolation buffer to digest cardiac tissue. After plating on laminin‐coated coverslips, cardiac myocytes were fixed with 4% paraformaldehyde for 5 min, and incubated with blocking/permeabilization buffer overnight (10% bovine calf serum and 0.2% Triton X‐100 in PBS). Subsequently, cardiac myocytes were incubated with primary antibodies in blocking buffer at 4°C overnight as follows: P‐S6 Ribosomal Protein^S235/236^, dilution 1:500 (catalog no. 2211; Cell Signaling Technology); RyR2, dilution 1:500 (catalog no. MA3‐916; Thermo Scientific); and PLN, dilution 1:500 (catalog no. ab2865, Abcam). RyR2 was localized using a mouse monoclonal antibody (clone C3‐33). For initial immunoblotting and immunocytochemistry, clone C3‐33 was obtained from Abcam (ab2827); following its discontinuation, the same clone was sourced from Thermo Fisher Scientific (MA3‐916) for STED imaging. The specificity and labelling patterns of both preparations were confirmed to be identical. After washing in blocking buffer, samples were incubated with secondary antibodies diluted 1:1000 for 2 h at room temperature: goat anti‐rabbit STAR635P (catalog no. 2‐0012‐007‐2; Abberior, Heidelberg, Germany) and goat anti‐mouse STAR580 (catalog no. 2‐0002‐005‐1; Abberior). Finally, mounting medium (ProLong Gold Antifade Mountant; Thermo Fisher Scientific) was used to embed the samples after washing in PBS. For imaging, a TCS SP8 STED system (Leica) with an HC PL APO C2S 100×/1.40 oil immersion objective (Leica) was used. Confocal images were acquired using the following parameters: pixel size: 116.34 nm × 116.34 nm; pixel dwell time: 400 ns; scanning speed: 600 Hz; 16× line averaging; fluorophore excitation by a white‐light laser at 635 and 580 nm; and fluorescence detection between 650–700 nm and 600–630 nm, respectively. For acquisition of STED images, an additional STED depletion laser at 775 nm was used. Images were acquired at a resolution of 16.61 × 16.61 nm. For optimal signal resolution of STAR635P and STAR580, the STED laser beam was adjusted accordingly. Fiji (https://imagej.net/Fiji) was used for raw image processing and line profiles were plotted in Prism, version 7.0 (GraphPad Software Inc., San Diego, CA, USA).

### Electron microscopy

Adult rat ventricular myocytes were attached to poly‐l‐lysine‐coated sapphire discs provided by Engineering Office M. Wohlwend GmbH (Sennwald, Switzerland) and placed in a culture dish containing Medium 199 (catalog no. M7528; Sigma‐Aldrich) supplemented with 100 units mL^−1^ penicillin, 100 µg mL^−1^ streptomycin and 10 µg mL^−1^ laminin (catalog no. L2020; Sigma‐Aldrich). The sample was incubated for 1 day. The discs were then dipped in a solution of 20% polyvinylpyrrolidone (Sigma‐Aldrich) in PBS, after which they were subjected to high‐pressure freezing using an HPM 010 instrument (Bal‐Tec, Balzers, Liechtenstein). During high‐pressure freezing, the sapphire disc containing the cells was sandwiched between two aluminium planchettes (Engineering Office M. Wohlwend GmbH). The 0.1 mm deep aluminium carrier was placed over the cells and the flat surface of the 0.3 mm thick carrier was placed beneath the sapphire disc. Freeze substitution of the cardiomyocytes was then performed using a freeze substitution device (EM‐AFS2; Leica Microsystems, Vienna, Austria). The freeze substitution solution contained 0.2% (w/v) osmium, 0.1% (w/v) uranyl acetate and 5% (v/v) water in dry acetone. The samples were substituted at −90°C for 2 h. The temperature was then increased at a rate of 5°C h^−1^ until reaching 20°C and incubated for 1 h at 20°C. The samples were then rinsed with acetone, followed by stepwise infiltration (30%, 50%, 75% and 100%) in Spurr's resin (Serva, Heidelberg, Germany). Resin polymerization was then performed at 60°C for 48 h. Sectioning was performed at 45° using a Diatome diamond ultra‐knife on a UC6 microtome (Leica). Thin sections (70 nm) were collected on Formvar‐coated copper slot grids. The grids were stained for 30 s with Reynolds' lead citrate, then for 1 min with 4% uranyl acetate in water and, finally, for a further 30 s with Reynolds' lead citrate to visualize the ribosomes effectively. The sections were imaged using a JEM‐1400 electron microscope (JEOL, Tokyo, Japan), operating at 80 kV.

### Immunoblotting

Cultured cells were lysed in RIPA buffer consisting of 50 mm Tris, pH 7.5, 150 mm NaCl, 1% Triton X‐100 and 1% SDS, which was supplemented with protease inhibitor cOmplete ULTRA (catalog no. 05892791001; Roche, Basel, Switzerland) phosphatase inhibitor PhosSTOP (catalog no. 04906837001; Roche). Lysates were cleared by centrifugation at 4°C for 10 min at 20,000 *g*. Lysate protein concentration was determined using DC Protein Assay Kit II (catalog no. 5000112; Bio‐Rad). Equivalent amounts of protein were brought up to similar volume, mixed with Laemmli Sample Buffer (catalog no. 161‐0747; Bio‐Rad) and 2‐mercaptoethanol (catalog no. M6250; Sigma‐Aldrich) and boiled at 95°C for 5 min. Samples were separated on SDS‐PAGE gels and transferred to Immobilon‐P transfer membranes (catalog no. IPVH00010; Merck Millipore, Burlington, MA, USA). The following antibodies were used to probe the membranes: Ryanodine Receptor (catalog no. ab2827; Abcam; dilution 1:1000), SERCA2 (catalog no. sc‐8095, N‐19; Santa Cruz Biotechnology; dilution 1:1000), KDEL (catalog no. 10C3, ADI‐SPA‐827, ENZO Life Sciences; dilution 1:2000–5000), Phospho‐S6 Ribosomal Protein Ser^235/236^ (catalog no. D57.2.2E, 4858; Cell Signaling Technology; dilution 1:5000) and S6 Ribosomal Protein (catalog no. 54D2, 2317; Cell Signaling Technology; dilution 1:1000). Ponceau solution was prepared with Ponceau BS (catalog no. B6008; Sigma‐Aldrich). Specificity for primary antibodies was validated through the observation of expected molecular weights in western blots and distinct, established subcellular localization patterns in cardiac myocytes.

### Statistical analysis

Statistical analysis was performed using Prism, version 7.0 (GraphPad Software Inc.). An unpaired two‐tailed *t* test was used to compare two groups. Data are presented as the mean ± SD. Sample sizes (*n*) for each experiment represent independent biological replicates (e.g. independent cell isolations) and are explicitly stated in figure legends. *P* < 0.05 was considered statistically significant. Exact *P* values are reported to three significant figures, except for *P* < 0.001.

## Results

To investigate developmental changes in the distribution and functional specialization of the ER (protein synthesis and secretion) and SR (Ca^2^^+^ homeostasis and EC coupling), primary ventricular myocytes were isolated from neonatal (1–3‐day‐old) and adult (8‐week‐old) rats (Sengupta, 2013). Protein abundance and subcellular localization of key regulators of these processes were then assessed (Fig. 1*A*). Cardiac ryanodine receptor 2 (RyR2), the primary Ca^2^^+^ release channel of the junctional SR, and sarco/endoplasmic reticulum Ca^2^^+^‐ATPase (SERCA2), the major pump responsible for Ca^2^^+^ reuptake into the SR, were used as representative markers of Ca^2^^+^ homeostasis and EC coupling. The chaperones 78 kDa glucose‐regulated protein (GRP78) and GRP94, together with protein disulfide isomerase family A member 6 (PDIA6), were used as markers of protein folding, processing, and quality control, whereas phosphorylated and total ribosomal protein S6 (RPS6) were examined as markers of ribosome abundance and translational activity. Compared with adult cardiac myocytes, neonatal myocytes exhibited higher relative expression of ER/SR‐resident protein quality control components, together with increased relative ribosome abundance, whereas adult cardiac myocytes exhibited higher relative expression of Ca^2^^+^ handling proteins (Fig. 1*B*). Taken together, these findings indicate that cardiac myocyte maturation is accompanied by a functional shift, in which immature cells exhibit a relative enrichment of ER‐associated processes, whereas adult myocytes exhibit a relative enrichment of SR‐associated functions to meet increased contractile demand.

**Figure 1:**
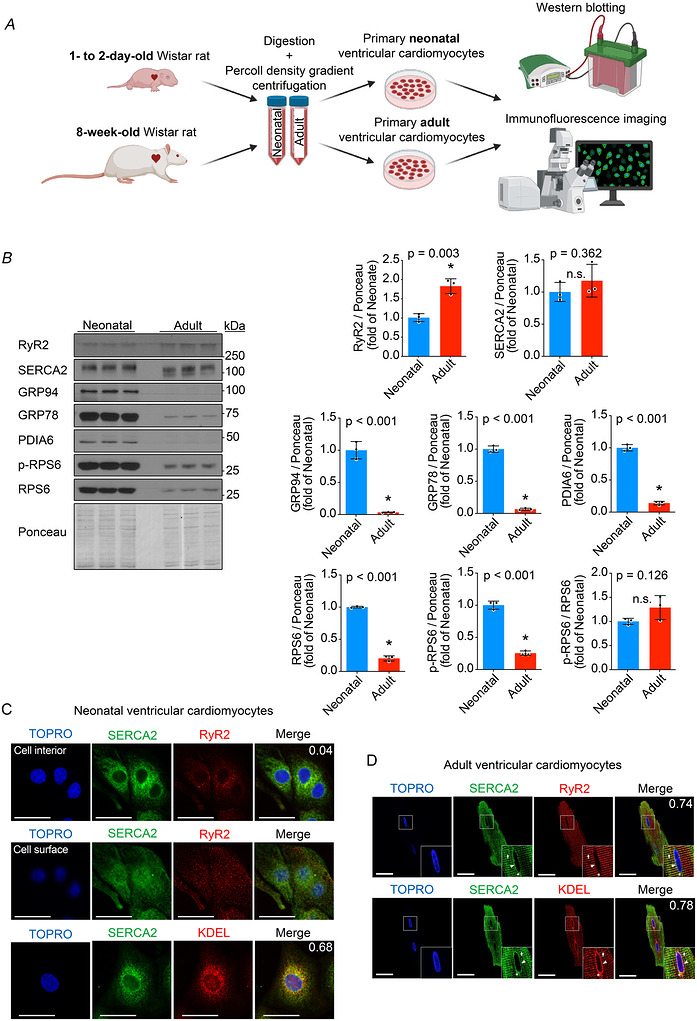
Postnatal maturation of cardiac myocytes is characterized by the expansion of the sarcoplasmic reticulum and a relative decrease in endoplasmic reticulum markers *A*, cardiac myocytes were isolated from neonatal and adult rat hearts and subjected to immunoblotting and immunocytofluorescence. *B*, immunoblots and respective quantification of neonatal and adult rat ventricular cardiac myocytes for markers of Ca^2+^ homeostasis and excitation–contraction coupling (RyR2 and SERCA2), protein processing and quality control (GRP94, GRP78 and PDIA6) and ribosome quantity and activity (RPS6 and p‐RPS6^S235/236^). Data are presented as the mean ± SD with all individual data points plotted. For neonatal samples, *n* = 3 independent cell preparations (each preparation represents a pool of ∼100 neonatal hearts); for adult samples, *n* = 3 independent cell preparations (each preparation represents an individual adult heart). Exact *P* values for all comparisons are shown. The Ponceau‐stained membrane in (*B*) is shown as a continuous, unspliced image; the apparent straight line between bands reflect the original membrane/background and does not indicate splicing or removal of lanes. *C*, coimmunofluorescence labelling of neonatal rat ventricular myocytes with antibodies to the Ca^2+^‐ ATPase of the SR (SERCA) and either the cardiac ryanodine receptor (RyR2) or an antibody that specifically recognizes proteins containing the KDEL sequence, used as an ER marker. *D*, coimmunofluorescence labelling of adult rat ventricular myocytes as described in (*C*). TOPRO, nuclei; scale bars = 20 µm.

### The sarcoplasmic reticulum and endoplasmic reticulum of cardiac myocytes form interconnected membrane domains with distinct but overlapping functions

To distinguish between ER‐ and SR‐associated functions within the cardiac myocyte membrane network, we performed immunofluorescence labelling in neonatal and adult rat ventricular myocytes, targeting the EC coupling proteins RyR2 and SERCA2, as well as the tetrapeptide KDEL, a canonical retention signal for ER‐resident proteins in other cell types (Glembotski, 2012). In neonatal cardiac myocytes, SERCA2 displayed diffuse labelling of a perinuclear membrane network, consistent with the distribution of the ER in other cell types (Fig. 1*C*). By contrast, RyR2 localized to small perinuclear clusters and appeared as distinct punctate structures towards the cell surface when the focus was shifted above the nucleus (Fig. 1*C*), consistent with previous reports showing that RyR2 is initially concentrated near the plasma membrane in immature cardiac myocytes and relocates towards the cell interior during maturation (Dan et al., 2007). The distinct localization patterns of SERCA2 and RyR2 reflect the developing organization of the membrane network and mark sites of nascent junctions with the sarcolemma. Although structural T‐tubules do not fully develop in rodents until later in the postnatal period, these peripheral RyR2 clusters represent the early spatial organization required for the eventual establishment of EC coupling machinery. KDEL staining in neonatal cardiac myocytes colocalized with SERCA2 (Fig. 1*C*), indicating that protein quality control and Ca^2+^‐handling functions overlap within a predominantly ER‐like perinuclear membrane network. In adult cardiac myocytes, confocal microscopy revealed two distinct but interconnected membrane domains: (1) a perinuclear ER‐like network, marked by overlapping SERCA and KDEL staining and continuous with the nuclear envelope, and (2) a peripheral striated SR, characterized by overlapping SERCA and RyR2 staining with weaker KDEL signal (Fig. 1*D*). Although these membrane networks appear functionally overlapping and interconnected by confocal microscopy, we hereafter refer to the perinuclear network as ER and the striated network as SR for clarity. In adult cardiac myocytes, RyR2 was absent from the nuclear envelope (Fig. 1*D*, arrowhead) and the ER‐like perinuclear membrane network (Fig. 1*D*, asterisk; KDEL staining), but localized predominantly to the SR (arrow) (Fig. 1*D*). Thus, RyR2 serves as a marker to distinguish ER from SR in adult cardiac myocytes. In contrast, KDEL, although commonly used as an ER marker in other cell types, is not restricted to the perinuclear ER‐like network in adult cardiac myocytes and is also detected throughout the striated SR.

In non‐myocytes, the ER is the site where protein secretion via the classical pathway is initiated (Doroudgar & Glembotski, 2011). In the classical secretory pathway, proteins are synthesized on ER‐bound ribosomes, translocated into the ER, transported to the Golgi and ultimately secreted when secretory vesicles fuse with the plasma membrane (Meyer & Doroudgar, 2020). To further define the functions of the ER and SR in cardiac myocytes, we assessed whether both networks participate in classical protein processing for secretion, a role typically attributed to the ER in other cell types. We first mapped the localization of secretory pathway components relative to the ER and SR in neonatal and adult ventricular myocytes. Both the cis‐Golgi marker GM130 and the trans‐Golgi marker TGN38 were closely associated with the perinuclear ER subcompartment in both neonatal and adult cardiac myocytes (Fig. 2). By contrast, CD63, a marker of late endosomes and related secretory organelles (Kobayashi et al., 2000), was distributed throughout the cell, suggesting that endosomes can move freely within cardiac myocytes after formation at the ER‐associated Golgi (Fig. 2). Together, these findings demonstrate the presence of a perinuclear ER and a peripheral SR network in adult differentiated cardiac myocytes. Although the classical secretory machinery is concentrated at the perinuclear ER, consistent with its role as a primary hub, its presence within the cell periphery suggests that the SR may also contribute to specialized protein processing (Blackwood, Azizi et al., 2019; Jin et al., 2016; Tadimalla et al., 2008).

**Figure 2:**
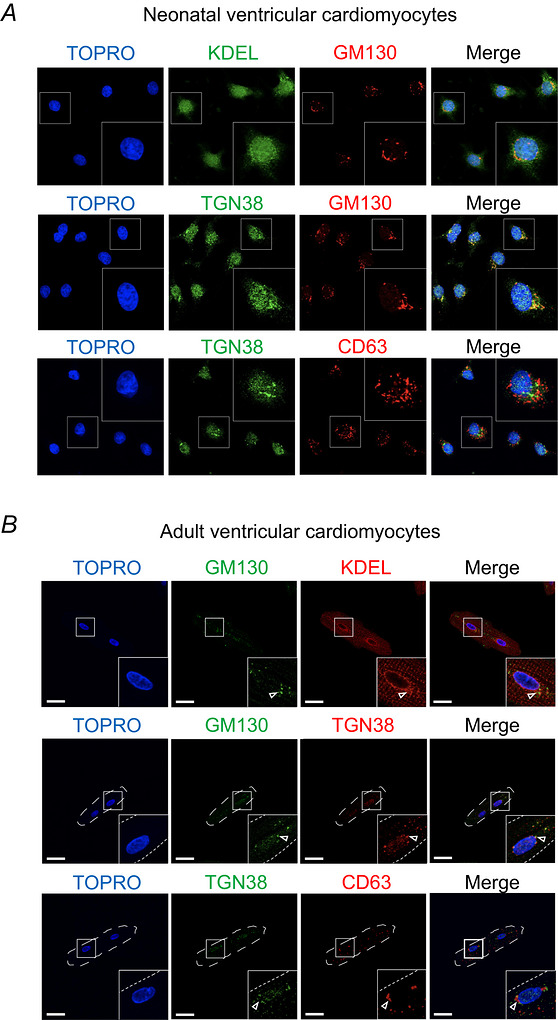
The classical secretory machinery and Golgi apparatus are predominantly localized to the perinuclear ER in neonatal and adult cardiac myocytes *A*, coimmunofluorescence labelling of neonatal rat ventricular myocytes with antibodies to the ER marker KDEL, the cis‐Golgi marker GM130, the trans‐Golgi marker TGN38 and the exosome marker CD63. *B*, coimmunofluorescence labelling of adult rat ventricular myocytes as described in (*A*). TOPRO, nuclei; scale bars = 20 µm.

### Ribosomes localize to both the sarcoplasmic and endoplasmic reticulum in adult cardiac myocytes

We next sought to identify the membrane networks associated with protein synthesis during cardiac myocyte maturation. To this end, we performed immunofluorescence co‐localization studies in neonatal and adult rat cardiac myocytes, labelling p‐RPS6, a marker associated with ribosomal activation and translational activity, together with RyR2, SERCA or KDEL (Fig. 3). Phosphorylation of RPS6 correlates with ribosome activation and translation of specific mRNA transcripts in the heart (Bohlen et al., 2021; Górska et al., 2021; Hofmann et al., 2023, Hofmann, Serafin, et al., 2024; Peterson & Schreiber, 1998), including transcripts involved in cell cycle progression, protein synthesis, and specialized processes regulating cardiac function (Górska et al., 2021; Hofmann, Serafin et al., 2024; Jefferies et al., 1997; Peterson & Schreiber, 1998). In neonatal rat cardiac myocytes, p‐RPS6 was distributed throughout the cell, with particularly intense staining at the perinuclear ER (Fig. 3*A*). By contrast, in adult rat cardiac myocytes, p‐RPS6 was localized to both the perinuclear ER and the striated SR (Fig. 3*B*). These findings indicate that, during maturation, the ribosomal machinery becomes spatially organized across both ER and SR domains, consistent with a distributed sarco/endoplasmic reticulum protein synthesis network in adult cardiac myocytes.

**Figure 3:**
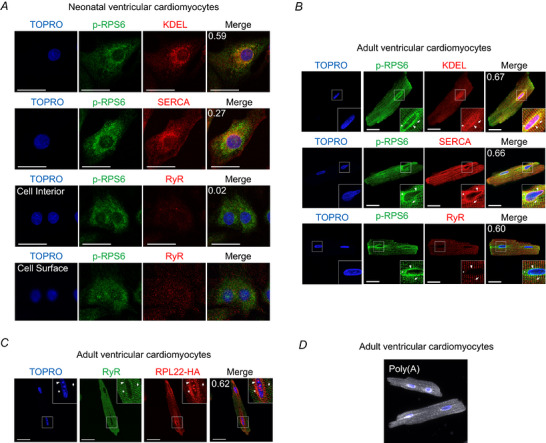
Ribosomes are localized throughout the neonatal and adult ventricular myocyte ER and SR membrane network *A*, coimmunofluorescence labelling of neonatal rat ventricular myocytes or (*B*) adult rat ventricular myocytes with antibodies to the S6 ribosomal protein phosphorylated at Ser^235/236^ (p‐RPS6) and either an antibody that specifically recognizes proteins containing the KDEL sequence, used as an ER marker, the Ca^2+^‐ATPase of the SR (SERCA), or the cardiac ryanodine receptor (RyR2). *C*, coimmunofluorescence labelling of isolated adult ventricular myocytes from RiboTag mice, expressing hemagglutinin (HA)‐tagged ribosomal protein RPL22, which is incorporated into actively translating polyribosomes. TOPRO, nuclei; scale bars = 20 µm. *D*, mRNA fluorescence in situ hybridization (FISH) of poly(A)‐mRNAs in adult rat cardiac myocytes. For all representative high‐resolution images, spatial intensity profiling (quantified overlap) was performed to demonstrate the precise peak alignment of ribosomal markers (p‐RPS6/HA‐RPL22) and mRNA with the longitudinal SR, junctional SR and perinuclear ER membranes.

Additionally, p‐RPS6 localized to the distal ends of cardiac myocytes near the intercalated discs, as previously described (Bogdanov et al., 2021; Lewis et al., 2018). RPS6 is a component of the 40S ribosomal subunit (Yusupova & Yusupov, 2014). To further validate the localization of ribosomes within the adult cardiac myocyte membrane network, we isolated adult ventricular myocytes from RiboTag mice, which we previously used to study cardiac myocyte protein synthesis under stress (Doroudgar et al., 2019; Hofmann, Serafin et al., 2024). RiboTag mice express hemagglutinin (HA)‐tagged ribosomal protein RPL22, a component of the 60S ribosomal subunit incorporated into actively translating polyribosomes (Doroudgar et al., 2019; Sanz et al., 2009; Wilson & Doudna Cate, 2012). In this model, expression of the HA‐tagged RPL22 is driven by a cell‐type‐specific promoter targeting cardiac myocytes. Similar to p‐RPS6, HA‐RPL22 localized to both the ER and SR in adult ventricular myocytes, confirming that the observed ribosome staining pattern is consistent across ribosomal subunits, antibodies, and species (Fig. 3*C*). If ribosomes bound to the ER and SR are actively translating, mRNAs should generally localize to the same subcellular regions. To test this, we performed mRNA fluorescence in situ hybridization (FISH) to visualize poly(A)‐mRNA distribution in cardiac myocytes (Fig. 3*D*). Similar to ribosomes, poly(A)‐mRNAs localized to the perinuclear ER‐like region and peripheral striated SR network in adult cardiac myocytes (Fig. 3*D*). While poly(A)‐mRNA FISH provides a global visualization of the transcriptome rather than identifying specific SR‐resident transcripts, the close spatial association of total mRNA and active ribosomes with the SR suggests a capacity for localized translation within this peripheral network.

To map the sites of active protein synthesis, we utilized acute puromycin pulsing. Puromycin, an aminoacyl‐tRNA structural analogue, incorporates into elongating peptide chains and can be detected with an anti‐puromycin antibody. Neonatal and adult cardiac myocytes were treated with 0.5 µg mL^−1^ puromycin and rapidly fixed to prevent dissociation of the puromycin‐labelled polypeptides (Salic, 2012). In neonatal cardiac myocytes, puromycin‐labelled nascent polypeptides were distributed throughout the cell, with the highest signal intensity concentrated in the perinuclear region (Fig. 4*A*). By contrast, adult cardiac myocytes displayed a distinct striated pattern of puromycin‐labelled nascent polypeptides throughout the cell periphery (Fig. 4*B*). This pattern closely mirrors the striated organization of the peripheral SR network observed in our RyR2 and SERCA2 immunofluorescence (Fig. 1*D*), suggesting that the SR becomes a major site of active translation as cardiac myocytes mature. The specificity of the puromycin signal was confirmed by cycloheximide co‐treatment, which abolished the labelling in both developmental stages. These findings are consistent with recent reports describing localized translation of specific mRNAs at distinct subcellular regions (Bogdanov et al., 2021; Douvdevany et al., 2024; Lewis et al., 2018; Scarborough et al., 2021) and support the concept that the SR represents a specialized site of translation within adult cardiac myocytes.

**Figure 4:**
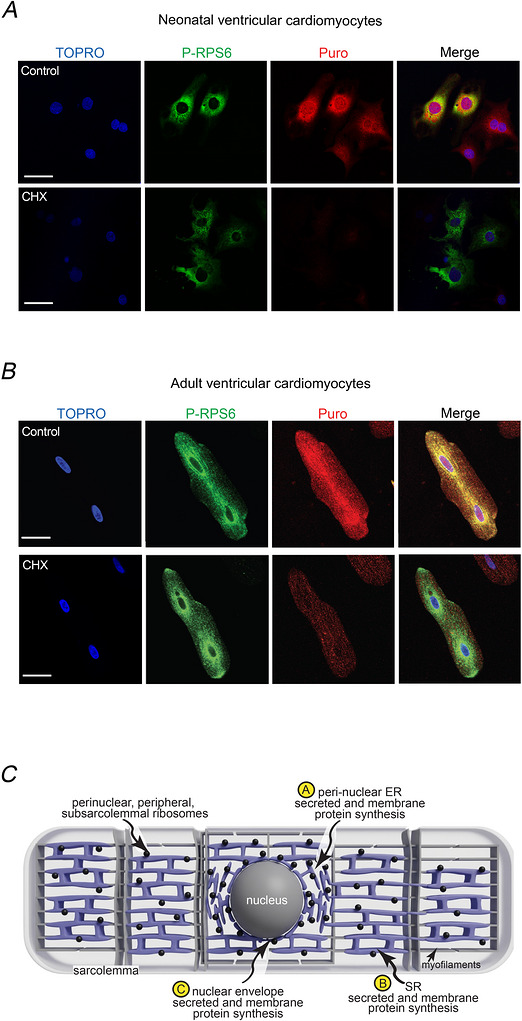
Protein synthesis is localized near the SR and ER in cardiac myocytes *A*, coimmunofluorescence labelling of neonatal rat ventricular myocytes or (*B*) adult rat ventricular myocytes with antibodies to the S6 ribosomal protein phosphorylated at Ser^235/236^ (p‐RPS6) and an antibody that specifically recognizes puromycin incorporated into nascent proteins (Puro). Specificity of the puromycin signal was validated by co‐treatment with cycloheximide (CHX), a potent inhibitor of mRNA translation, which abolished nascent polypeptide labelling in both developmental stages. Scale bars: 50 µm (*A*), 20 µm (*B*). *C*, schematic of the sarco/endoplasmic reticulum network in a cardiac myocyte, depicting the proposed relationships between the SR (*A*), the perinuclear ER (*B*), and nuclear envelope (*C*). The localization of secreted and membrane protein synthesis to the nuclear envelope, perinuclear ER and the SR, depicted as a contiguous membranous system, is shaded purple.

### Superresolution and ultrastructural imaging confirm ribosome association with the SR in cardiac myocytes

To further examine ribosome localization to the SR in adult cardiac myocytes, we performed transmission electron microscopy to define ribosome localization at ultrastructural resolution. Transmission electron microscopy of high‐pressure frozen cells revealed membrane‐bound ribosomes associated with perinuclear membranes (ER) (Fig. 5*A*), the peripheral SR network (Fig. 5*B*), SR membranes adjacent to T‐tubules (Fig. 5*C*), and the subsarcolemmal SR at the terminal sarcolemma of the intercalated disc (Fig. 5*D*), thereby confirming the ribosome distribution observed by confocal microscopy.

**Figure 5:**
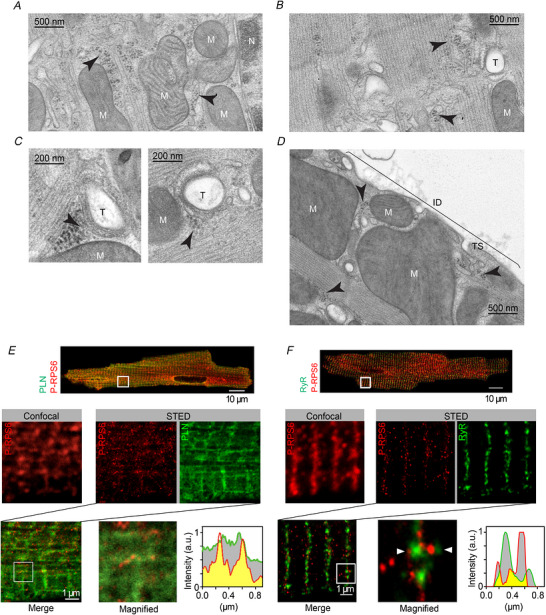
Ribosomes colocalize with the SR Representative electron micrographs of adult rat ventricular myocytes show (*A*) ribosome (arrowheads) association with perinuclear membranes (ER), (*B*) peripheral membranes (longitudinal, free SR), (*C*) T‐tubules (near junctional SR) and (*D*) in the subsarcolemmal region, specifically at the terminal sarcolemma (TS) of intercalated disc (ID). M, mitochondria; T, T‐tubules; N, nucleus. *E*, coimmunofluorescence imaging of phosphorylated ribosomal protein S6 (p‐RPS6; red) and phospholamban (PLN; green) or (*F*) ryanodine receptor type 2 (RyR2) in isolated mouse ventricular myocytes using both confocal microscopy and STED nanoscopy. Zoomed‐in images demonstrating the local association of p‐RPS6 with individual PLN or RyR2. White rectangles indicate the orientation of the signal intensity profiling. Colocalized signals are indicated in yellow. White boxes in (*A*) and (*B*) indicate magnified regions. Scale bars: 10 µm (cell overview), 1 µm (confocal, STED) and 200 nm (magnified view). STED, stimulated emission depletion microscopy.

Although electron microscopy demonstrated a clear association of ribosomes with membrane systems throughout the cardiac myocyte, it could not distinguish between different membrane networks and therefore could not definitively demonstrate that ribosomes are directly associated with the SR. Similarly, our confocal co‐localization studies could not resolve direct membrane association because of the diffraction limit of conventional light microscopy. Superresolution microscopy using STED overcomes this limitation, offering diffraction‐unlimited resolution down to ∼20 nm, which approaches the dimensions of a eukaryotic ribosome (Vicidomini et al., 2018; Yusupova & Yusupov, 2014). To determine whether ribosomes associate with distinct SR domains, we co‐stained adult cardiac myocytes with p‐RPS6 (ribosome marker) and either phospholamban (PLN; longitudinal SR) or RyR2 (junctional SR) using STED microscopy. Co‐staining with PLN demonstrated close spatial association of ribosomes with the longitudinal SR. By contrast, co‐staining with RyR2 showed that ribosomes were closely apposed to, but excluded from, RyR2‐rich junctional SR domains (Fig. 5*E* and *F*), indicating that active ribosomes preferentially localize to the longitudinal SR membrane network in adult cardiac myocytes.

Taken together, our findings demonstrate that mature cardiac myocytes contain distinct yet interconnected ER and SR membrane domains that perform specialized but overlapping functions. The perinuclear membrane system, which is continuous with the nuclear envelope, performs ER‐like functions, including protein secretion via the classical secretory pathway, whereas the peripheral network carries out SR‐associated functions, such as calcium‐induced calcium release. Using complementary confocal, electron, and superresolution imaging approaches, we demonstrate that ribosomes localize to both the ER and the longitudinal SR. Together with the localization of poly(A) mRNA and sites of active protein synthesis, these findings support the concept that the adult sarco/endoplasmic reticulum constitutes a spatially organized protein synthesis network.

## Discussion

The ER is a primary site of protein synthesis, responsible for the synthesis, folding, modification, and transport of proteins essential for excitable cells, including ion channels, receptors, calcium‐handling proteins, and secreted proteins. In striated muscle cells, a specialized membrane network distinct from the ER, the SR, surrounds the myofilaments and regulates intracellular calcium release required for contraction. However, the precise localization, protein production functions and expression profiles of the ER and SR in cardiac myocytes remain unclear (Doroudgar & Glembotski, 2013). Using multiple imaging approaches, including superresolution microscopy, we demonstrate that adult cardiac myocytes contain two functionally distinct yet interconnected membrane domains: a perinuclear ER domain and a peripheral SR domain, similar to the organization observed in skeletal muscle cells (Doroudgar & Glembotski, 2013; Sleiman, McFarland et al., 2015; Wu & Bers, 2006).

Cardiac contraction is triggered by Ca^2+^ release from the SR in response to an action potential, a process known as EC coupling. The components of EC coupling are under tight spatial and temporal regulation and mature during development (Legato, 1979, 2012; Olivetti et al., 1980; Sedarat et al., 2000; Smolich, 1995; Tibbits et al., 2002; Ziman et al., 2010). Postnatal development of cardiac myocytes is accompanied by reorganization of cellular architecture, supporting increased mitochondrial and myofibrillar mass as well as SR maturation (Piquereau et al., 2010). Indeed, analysis of global transcriptomic trends during mammalian heart development indicates that, although sarcomeric and mitochondrial genes become the most abundant transcripts in the adult, the absolute number of translational components does not scale linearly with total cell volume (Brawand et al., 2011). This suggests that the apparent downregulation of ER and ribosomal markers we observed in adult myocytes probably reflects a ‘dilution’ effect within the total proteome as the cytoplasmic space is filled with well‐aligned myofilaments and mitochondria. Consistent with this, we observed an expansion of the SR from neonatal to adult myocytes. In adults, the SR extends throughout much of the cellular periphery, whereas the ER remains confined to a smaller perinuclear region. We propose that this developmental transition represents a redistribution of the translational machinery from a concentrated perinuclear hub in neonatal myocytes to a distributed sarco/endoplasmic reticulum network in adult myocytes, supporting localized protein synthesis across the markedly expanded cytoplasmic volume. Given the extraordinary size and highly organized architecture of adult cardiac myocytes, such a distributed protein synthesis network may reduce the need for long‐distance intracellular protein trafficking while facilitating local proteostasis throughout the contractile apparatus.

Our imaging data suggest that classical secretory pathway activity is concentrated primarily within the perinuclear ER, where the density of quality control chaperones and Golgi markers is highest. Although this supports a model where the initiation of secretion is a predominantly perinuclear event, our observations also revealed the presence of secretory machinery in the cell periphery, albeit at a lower relative abundance. The greater perinuclear immunofluorescence intensity may reflect the high density of ER membranes within a relatively small volume, whereas translational machinery distributed across the extensive peripheral SR produces a comparatively diffuse signal despite its substantial overall abundance. This aligns with the findings reported by Bogdanov et al. (2021) and suggests that, although the ER exhibits greater specialization for protein secretion, the SR may maintain a capacity for specialized or localized secretory functions. Together, these findings support a model in which the ER serves as the primary hub for the classical secretory pathway, whereas the SR provides a distributed platform for localized protein synthesis within the contiguous sarco/endoplasmic reticulum network.

Two models have been proposed for the relationship between the ER and SR in cardiac myocytes and their roles in protein synthesis (Doroudgar & Glembotski, 2013), largely based on studies in skeletal muscle. In the first model, protein synthesis and secretion are confined to the nuclear envelope and perinuclear ER, defining a network specialized for protein processing, distinct from a ‘smooth’ SR that does not participate in protein synthesis but regulates calcium. In the second model, protein synthesis, folding, modification and transport occur throughout a contiguous membrane network comprising both a perinuclear ER and a peripheral rough SR. Our data support the second model; however, certain functions, such as RyR2‐mediated Ca^2+^ release, remain specific to the SR. Thus, the ER and SR appear to represent molecularly specialized domains within a continuous sarco/endoplasmic reticulum network rather than entirely separate membrane systems. It is important to note that established models of cardiac protein biogenesis emphasize a centralized synthesis route for SR‐resident proteins, such as SERCA2a, phospholamban, and junctin, originating in the juxtanuclear rough ER and the nuclear envelope (He et al., 2020; Sleiman, McFarland et al., 2015). These studies highlight a sophisticated trafficking network, including the NEST (Nuclear Envelope to SR along T‐tubules) pathway, that utilizes microtubules to transport newly synthesized proteins from the nuclear axis to the cell periphery.

Recent studies also show that cardiac myocytes localize specific mRNAs to distinct subcellular regions, where they associate closely with ribosomes, consistent with localized translation (Bogdanov et al., 2021; Douvdevany et al., 2024; Lewis et al., 2018; Scarborough et al., 2021). This process appears to depend, at least in part, on microtubule‐associated transport (Scarborough et al., 2021). The localization of mRNA to the SR probably involves specific *cis‐*acting elements within the mRNA transcripts that facilitate transport via the microtubule network, analogous to the ‘zipcode’ targeting mechanisms observed in neurons (Holt & Schuman, 2013). Given the high metabolic turnover and the large size of adult cardiac myocytes, such localized translation at the SR could minimize the energetic cost of long‐distance protein trafficking and facilitate the rapid replacement of Ca^2+^‐handling machinery at the cell periphery. Although the intracellular membrane system of cardiac myocytes is known to be essential for translation and processing of key proteins, including secreted and membrane proteins such as ion channels and cell surface receptors, the distinctions between the ER and SR, and whether the SR participates in protein synthesis and processing, have remained unclear (Doroudgar & Glembotski, 2013).

Our findings indicate that the SR in adult cardiac myocytes represents a previously unrecognized site of localized translation. Although definitive evidence will require direct visualization of the translation of SR‐localized mRNAs, our observation of active ribosomes, poly(A) mRNA, and nascent protein synthesis localized to the SR network, particularly in the mature, SR‐dominant state, provides strong support for this model. This is consistent with the paradigm of polarized cells, such as neurons, which utilize localized translation to maintain protein homeostasis at distal functional sites. Together, our findings support a model in which maturation of the cardiac myocyte is accompanied by the development of a spatially organized sarco/endoplasmic reticulum protein synthesis network that complements the well‐established role of the SR in Ca^2+^ handling and enables localized proteostasis throughout the adult cell.

The specialized structural and functional segregation of the ER and SR in the healthy adult heart appears to be highly susceptible to remodelling during pathology. In chronic conditions such as hypertrophy or heart failure, the maintenance of this distinct, distributed synthesis network may be compromised as the cell prioritizes global proteostasis over specialized function. We and others have previously demonstrated that ischemia and pressure overload specifically activate the ATF6 branch of the ER stress response (Blackwood, Hofmann et al., 2019; Doroudgar et al., 2009; Hofmann, Aghajani et al., 2024; Martindale et al., 2006; Minamino et al., 2010; Thuerauf et al., 2006). Although ATF6 serves a critical protective role by transcriptionally inducing chaperones to prevent protein misfolding (Blackwood et al., 2020), its activation also drives metabolic and structural shifts, such as the induction of the mTORC1 activator Rheb to regulate cardiac hypertrophy (Blackwood, Hofmann et al., 2019). This shift suggests that under pathological stress, the myocyte may transition towards a more ‘ER‐dominant’ phenotype, reminiscent of early postnatal stages, where the specialized protein synthesis and Ca^2+^‐handling subdomains of the SR are eclipsed by a globalized quality control and growth program. Understanding the balance between the mature, distributed SR network and the stress‐induced ER program is critical for identifying how proteostasis failure contributes to the transition from compensated hypertrophy to heart failure.

In summary, cardiac myocytes contain both ER and SR networks that perform overlapping yet distinct functions. In neonatal myocytes, the membrane compartment resembles the ER and decreases in relative abundance as the cells mature, whereas the SR becomes the dominant membrane network in adult myocytes. RyR2 distinguishes the two networks, being absent from the perinuclear ER but present in the peripheral SR. Although classical protein secretion occurs primarily from the ER, other functions, such as SERCA‐mediated Ca^2+^ reuptake, are shared between both networks. Using multiple imaging approaches, including superresolution microscopy, we show that, similar to the ER, the SR in adult cardiac myocytes associates with active ribosomes, contains localized mRNA, and supports active protein synthesis. Together, these findings establish the sarco/endoplasmic reticulum as a spatially organized protein synthesis network that integrates the specialized functions of the ER and SR in mature cardiac myocytes.

## Additional information

## Competing interests

The authors declare that they have no competing interests (financial or non‐financial) that could be perceived as prejudicial to the objectivity of the report.

## Author contributions

ChS, SD and CCG conceived and designed the research. ChS, SB, DU, BM, ClS, NH and JG performed the experiments. ChS, SB and DU analyzed the data. ChS, SD, CCG and MV interpreted the results of the experiments. ChS prepared the figures. ChS and SD drafted the manuscript. All authors edited and revised the manuscript critically for important intellectual content. All authors have approved the final version of the manuscript submitted for publication and agree to be accountable for all aspects of the work. All persons designated as authors qualify for authorship, and all those who qualify for authorship are listed.

## Funding

This work was supported by grants from the DZHK (German Centre for Cardiovascular Research) Excellence Program, the University of Arizona Health Sciences Career Development Award, the National Institutes of Health (HL170223), and the University of Arizona College of Medicine–Phoenix Translational Cardiovascular Research Center to SD. CCG was supported by the National Institutes of Health (HL178630, HL174576, and HL157027). ChS was supported by the German Heart Foundation (Deutsche Herzstiftung) and the German Cardiac Society. SD, ChS, and MV acknowledge support from the DZHK Partner Site Heidelberg/Mannheim.

## Generative AI statement

The authors declare that no generative artificial intelligence was used during the preparation of the manuscript.

## Supporting information


Peer Review History

## Data Availability

We confirm that no new datasets were generated or analyzed during the current study. All raw data supporting the findings of this study, including original western blot membranes and high‐resolution microscopy files, are available from the corresponding author upon reasonable request.
