# Supplementary material for: Development of a protein synthesis network at the sarco/endoplasmic reticulum in adult cardiac myocytes
Source: J Physiol. 2026 Aug 11;604(16):6925–42. doi: 10.1113/JP288658 (PMC13481018; doi:10.1113/JP288658)
Supplement: Supplementary file 1 [file TJP-604-6925-s001.pdf]

The Journal of Physiology

Development of a protein synthesis network at the sarco/endoplasmic reticulum in adult cardiac myocytes

Christoph Sandmann, Soeren Brandenburg, Dennis Uhlenkamp, Bianca Meyer, Clara Sandmann, Nicole Herzog, Julia Groß, Charlotta Funaya, Hugo Katus, Stephan E Lehnart, Christopher C Glembotski, Mirko Völkers, and Shirin Doroudgar
DOI: 10.1113/JP288658

Corresponding author(s): Shirin Doroudgar (sdoroudgar@arizona.edu)

The referees have opted to remain anonymous.

Review Timeline:

Submission Date:	15-Sep-2025
Editorial Decision:	21-Oct-2025
Revision Received:	07-Mar-2026
Editorial Decision:	02-Apr-2026
Revision Received:	03-May-2026
Accepted:	03-Jun-2026

Senior Editor: Bjorn Knollmann

Reviewing Editor: Michelle Collins

Transaction Report:

(Note: With the exception of the correction of typographical or spelling errors that could be a source of ambiguity, letters and reports are not edited. Depending on transfer agreements, referee reports obtained elsewhere may or may not be included in this compilation. Referee reports are anonymous unless the Referee chooses to sign their reports.)

Re: JP-RP-2025-288658 "Development of a protein synthesis network at the sarco/endoplasmic reticulum in adult cardiac myocytes" by Christoph Sandmann, Soeren Brandenburg, Dennis Uhlenkamp, Bianca Meyer, Clara Sandmann, Nicole Herzog, Julia Groß, Hugo Katus, Stephan E Lehnart, Christopher C Glembotski, Mirko Völkers, and Shirin Doroudgar

Dear Dr Doroudgar,

Thank you for submitting your manuscript to The Journal of Physiology. It has been assessed by a Reviewing Editor and by 2 expert referees and we are pleased to tell you that it is potentially acceptable for publication following satisfactory major revision.

Please advise your co-authors of this decision as soon as possible.

The referee reports are copied at the end of this email.

Please address all the points raised and incorporate all requested revisions or explain in your Response to Referees why a change has not been made. We hope you will find the comments helpful and that you will be able to return your revised manuscript within 2 months. If your article is NOT for a Special Issue, you may have 9 months to revise. If you require an extension, please contact journal staff: jp@physoc.org. Please note that this letter does not constitute a guarantee for acceptance of your revised manuscript.

Your revised manuscript should be submitted online using the link in your Author Tasks: Link Not Available. This link is accessible via your account as Corresponding Author; it is not available to your co-authors. If this presents a problem, please contact journal staff (jp@physoc.org). Image files from the previous version are retained on the system. Please ensure you replace or remove any files that are being revised.

If you do not wish to submit a revised version of your manuscript, you must inform our journal staff (jp@physoc.org) or reply to this email to request withdrawal. Please note that a manuscript must be formally withdrawn from the peer review process at one journal before it may be submitted to another journal.

TRANSPARENT PEER REVIEW POLICY: To improve the transparency of its peer review process, The Journal of Physiology publishes online as supporting information the peer review history of all articles accepted for publication. Readers will have access to decision letters, including Editors' comments and referee reports, for each version of the manuscript, as well as any author responses to peer review comments. Referees can decide whether or not they wish to be named on the peer review history document.

ABSTRACT FIGURES: Authors are expected to use The Journal's premium BioRender account to create/redraw their Abstract Figures. Information on how to access this account is here: https://physoc.onlinelibrary.wiley.com/journal/14697793/biorender-access.

This will enable Authors to create and download high-resolution figures. If authors have used the free BioRender service, they can use the instructions provided in the link above to download a high-resolution version suitable for publication.

The link provided should only be used for the purposes of this submission. Authors will be charged for figures created on this account if they are not related to this manuscript submission.

LANGUAGE EDITING AND SUPPORT FOR PUBLICATION: If you would like help with English language editing, or other article preparation support, Wiley Editing Services offers expert help, including English Language Editing, as well as translation, manuscript formatting, and figure formatting at www.wileyauthors.com/eeo/preparation. You can also find resources for Preparing Your Article for general guidance about writing and preparing your manuscript at www.wileyauthors.com/eeo/prepresources.

RIGOUR AND REPRODUCIBILITY: We recommend authors consult The Journal's Rigour and Reproducibility requirements to ensure that their manuscript is suitable for publication: https://physoc.onlinelibrary.wiley.com/pb-assets/hub-assets/physoc/documents/TJP-Rigour-and-Reproducibility-Requirements.pdf

REVISION CHECKLIST:

Check that your Methods section conforms to journal policy: https://jp.msubmit.net/cgi-bin/main.plex?form_type=display_requirements#methods.

Check that data presented conforms to the statistics policy: https://jp.msubmit.net/cgi-bin/main.plex?form_type=display_requirements#statistics.

Upload a full Response to Referees file. To create your 'Response to Referees': copy all the reports, including any comments from the Senior and Reviewing Editors, into a Microsoft Word, or similar, file and respond to each point, using font or background colour to distinguish comments and responses and upload as the required file type.

Please upload two versions of your manuscript text: one with all relevant changes highlighted and one clean version with no changes tracked. The manuscript file should include all tables and figure legends, but each figure/graph should be uploaded as separate, high-resolution files.

You may also upload:

- 'Potential Cover Art' for consideration as the issue's cover image
- Appropriate Supporting Information (Video, audio or data set: see https://jp.msubmit.net/cgi-bin/main.plex?form_type=display_requirements#supp).

We look forward to receiving your revised submission.

If you have any queries, please reply to this email and we will be pleased to advise.

Yours sincerely,

Bjorn Knollmann
Senior Editor
The Journal of Physiology

REQUIRED ITEMS

- The reference list must be in alphabetical order, rather than numbered, to comply with our Journal format.

- Your manuscript must include a complete Additional Information section, including competing interests; funding; author contributions and acknowledgements.

- Please upload separate high-quality figure files via the submission form.

- You must upload original, uncropped western blot/gel images (including controls) if they are not included in the manuscript. This is to confirm that no inappropriate, unethical or misleading image manipulation has occurred. These should be uploaded as 'Supporting information for review process only'. Please label/highlight the original gels so that we can clearly see which sections/lanes have been used in the manuscript figures. For more information, see: https://physoc.onlinelibrary.wiley.com/hub/journal-policies#imagmanip.

- Please ensure that the Article File you upload is a Word file.

- Papers must comply with the Statistics Policy: https://jp.msubmit.net/cgi-bin/main.plex?form_type=display_requirements#statistics.

In summary:

- If n {less than or equal to} 30, all data points must be plotted in the figure in a way that reveals their range and distribution. A bar graph with data points overlaid, a box and whisker plot or a violin plot (preferably with data points included) are acceptable formats.

- If n > 30, then the entire raw dataset must be made available either as supporting information, or hosted on a not-for-profit repository, e.g. FigShare, with access details provided in the manuscript.

- 'n' clearly defined (e.g. x cells from y slices in z animals) in the Methods. Authors should be mindful of pseudoreplication.

- All relevant 'n' values must be clearly stated in the main text, figures and tables.

- The most appropriate summary statistic (e.g. mean or median and standard deviation) must be used. Standard Error of the

Mean (SEM) alone is not permitted.

- Exact p values must be stated. Authors must not use 'greater than' or 'less than'. Exact p values must be stated to three significant figures even when 'no statistical significance' is claimed.

- A Data Availability Statement is required for all papers reporting original data. This must be in the Additional Information section of the manuscript itself. It must have the paragraph heading 'Data Availability Statement'. All data supporting the results in the paper must be either: in the paper itself; uploaded as Supporting Information for Online Publication; or archived in an appropriate public repository. The statement needs to describe the availability or the absence of shared data. Authors must include in their statement: a link to the repository they have used, or a statement that it is available as Supporting Information; reference the data in the appropriate sections(s) of their manuscript; and cite the data they have shared in the References section. Whenever possible, the scripts and other artefacts used to generate the analyses presented in the paper should also be publicly archived. If sharing data compromises ethical standards or legal requirements then authors are not expected to share it, but must note this in their statement. For more information, see our Statistics Policy.

EDITOR COMMENTS

Reviewing Editor:

Comments for Authors to ensure the paper complies with the Statistics Policy:
Please define N values and report SD (rather than SEM). For data points where n<30, provide the data as supporting information. Provide precise p-values.

Comments to the Author:
The manuscript describes the distribution, maturation, and protein synthesis functions of the ER/SR networks in cardiomyocytes. This manuscript is well-written and the data are clearly presented. The work is recognized to be of broad interest to the field.

There are a few points raised by the reviewers that should be addressed to strengthen the manuscript, including quantification of confocal images, and consideration of whether reduced expression of ER proteins reflects redistribution as cell size increases with maturation.

Additional comments:

• Please define N values and report SD (rather than SEM).

• Please correct typographical errors in the figures and figure legends (e.g., fluorescence is written 'flourescence')

• Provide molecular weights for WBs.

• Figure 4 legend describe a panel C that is not found in the provided figure. CHX is not included in the figure legend.

• Scale bars should be added to the TEM figures in Fig 5A-D

• Please include an ethics statement and method of euthanasia.

Senior Editor:

Comments to the Author:
I concur with the Reviewing Editor. The manuscript is potentially interesting but would require a major revision to address the concerns raised in the review.

REFEREE COMMENTS

Referee #1:

Presented manuscript interrogates molecular differentiation and structural organization of the ER/SR during cardiac muscle development using biochemistry and imaging techniques. The authors demonstrate show that sarcoplasmic reticulum in adult cardiac myocytes possess some classical function of the ER. Namely, it binds active ribosomes and serves as a site of translation for some proteins such as RyR2. Please find my comments/suggestions below:

1) Line : 85 "Since most Ca2+ handling proteins reside in either the lumen of the SR or in the SR membrane [9,12], it is reasonable to posit that contractile Ca2+ handling proteins in cardiac myocytes are synthesized on ribosomes associated with the SR". Definitely not all of them according to PMID: 32353354 and PMID: 25640161. I suggest toning down this statement. Also, what is contractile Ca2+ handling proteins? Did you mean contractile and Ca2+ handling proteins

2) Is there any particular reason that different anti-RyR2 antibodies were used for STED and immunochemistry?

3) Line 206. Strictly speaking SERCA is not a channel.

4) Fig. 1.B. Could you please provide MWs for the westerns?

5) Quantification for imaging studies is missing which is a major concern

6) Does SR/ER functional overlap changes in anyway in cardiac disease?

Referee #2:

Sandmann et al. perform imaging and molecular studies in neonatal and adult rodent cardiomyocytes to assess the abundance and localization of classical secretory pathway and excitation-contraction coupling machinery and uncover distinct localization of protein synthesis machinery at the more classically-defined perinuclear endoplasmic reticulum as well as at the sarcoplasmic reticulum that is specialized for calcium cycling that suggest distinct but overlapping functions of the ER and SR in protein synthesis and translation. These studies support a model whereby protein biosynthesis, processing, and translation occur both at the classical perinuclear ER and the SR that form a contiguous membrane network for protein biosynthesis. These findings are important for the field and likely to be of broad interest to the readership of the J of Physiol. Some concerns with the manuscript and data interpretation are outlined below.

1. The concept that localized translation occurs in cardiomyocytes at the longitudinal SR , although perhaps beyond the scope of the current study and supported by the presence of actively translating ribosomes at the SR by multiple methods reported here, would require imaging of translation of mRNAs encoding resident SR proteins. Similarly, the concept the classical protein secretion occurs preferentially at the perinuclear ER compared to the SR is not fully supported by the current studies.

2. The authors demonstrate reduced expression of classical resident ER proteins anmd ribosomal markers in adult cardiomyocytes compared to neonatal cardiomyocytes concomitant with upregulation of RYR2 and conclude mature adult cardiomycoytes downregtulate secretory pathway machinery. Is it possible that the content of ribosomse/secretory pathway machinery etc. actually remain constant as CMs mature but become distributed in an orderly manner throughout the much larger cell as the cytoarchitecture is remodeled and the cytoplasmic space is filled with well-aligned myofilaments and mitochondria, thereby allowing for coordinated placement of translational machinery at ER and SR membranes throughout the adult cardiomyocyte to allow for efficient protein synthesis? Related, it would be interesting to see if relative myofilament protein and mitochonrdrial protein content are increased in adult vs. neonatal cardiomyoyctes. This may be challenging for myosin isoforms that switch but blotting for sarcomeric actin would be pretty straight forward. Also related, the immunofluoresence signal for translational machinery at the periphery/SR may be more diffuse as the translational machinery is distributed amongst a larger area whereas it is heavily concentrated in a smaller area in the perinuclear ER that although is significant may be somewhat misleading as to the relative abundance of ribosomes/translational machinery in the two regions.

3. Major title captions for figure legends for Figures 1 and 2 do not accurately summarize the presented data and/or findings and should be rewritten.

4. The claim that distinct localization patterns of SERCA2 and RyR2 in neonatal cardiomyocytes likely represent forming T-

tubules is unlikely to be the case as T-tubules do not begin forming until well over a week in rodent cardiomyocytes.

5. Fig legend 4C describes graphical schematic that is not present in the current figure (and may be worth including?)

6. TEM images in Figs 5A-D need scale bars.

7. Introduction discussion of junctional SR should include description of its close proximity to the T-tubules.

8.Typographical/grammatical errors: line 89, 111, 116, 139.

END OF COMMENTS

Dear Dr. Bjorn Knollmann, Reviewing Editor, and the Reviewers,

The authors appreciate the fair evaluation and valuable comments from the reviewers. Please find our point-by-point responses below.

Responses to Editor's Comments

REQUIRED ITEMS

- The reference list must be in alphabetical order, rather than numbered, to comply with our Journal format.

RESPONSE: The reference list has been reformatted in alphabetical order to comply with the Journal's format.

- Your manuscript must include a complete Additional Information section, including competing interests; funding; author contributions and acknowledgements.

RESPONSE: A complete Additional Information section has been added, including competing interests, funding, author contributions, and acknowledgements.

- Please upload separate high-quality figure files via the submission form.

RESPONSE: All figures have been uploaded separately as high-quality files via the submission form.

- You must upload original, uncropped western blot/gel images (including controls) if they are not included in the manuscript. This is to confirm that no inappropriate, unethical or misleading image manipulation has occurred. These should be uploaded as 'Supporting information for review process only'. Please label/highlight the original gels so that we can clearly see which sections/lanes have been used in the manuscript figures. For more information, see: https://physoc.onlinelibrary.wiley.com/hub/journal-policies#imagmanip.

RESPONSE: Original, uncropped western blot/gel images (including controls) have been uploaded as *Supporting information for review process only*.

- Please ensure that the Article File you upload is a Word file.

RESPONSE: The Article File has been uploaded in Microsoft Word format as requested.

- Papers must comply with the Statistics Policy: https://jp.msubmit.net/cgi-bin/main.plex?form_type=display_requirements#statistics.

RESPONSE: We have reviewed the Journal's Statistics Policy and confirm that all analyses and data presentation in the manuscript comply with these requirements.

In summary:

- If n {less than or equal to} 30, all data points must be plotted in the figure in a way that reveals their range and distribution. A bar graph with data points overlaid, a box and whisker plot or a violin plot (preferably with data points included) are acceptable formats.

- If n > 30, then the entire raw dataset must be made available either as supporting information, or hosted on a not-for-profit repository, e.g. FigShare, with access details provided in the manuscript.

- 'n' clearly defined (e.g. x cells from y slices in z animals) in the Methods. Authors should be mindful of pseudoreplication.

- All relevant 'n' values must be clearly stated in the main text, figures and tables.

- The most appropriate summary statistic (e.g. mean or median and standard deviation) must be used. Standard Error of the Mean (SEM) alone is not permitted.

- Exact p values must be stated. Authors must not use 'greater than' or 'less than'. Exact p values must be stated to three significant figures even when 'no statistical significance' is claimed.

RESPONSE: All figures and analyses have been updated to comply with the Journal's statistics requirements.

- A Data Availability Statement is required for all papers reporting original data. This must be in the Additional Information section of the manuscript itself. It must have the paragraph heading 'Data Availability Statement'. All data supporting the results in the paper must be either: in the paper itself; uploaded as Supporting Information for Online Publication; or archived in an appropriate public repository. The statement needs to describe the availability or the absence of shared data. Authors must include in their statement: a link to the repository they have used, or a statement that it is available as Supporting Information; reference the data in the appropriate sections(s) of their manuscript; and cite the data they have shared in the References section. Whenever possible, the scripts and other artefacts used to generate the analyses presented in the paper should also be publicly archived. If sharing data compromises ethical standards or legal requirements then authors are not expected to share it, but must note this in their statement. For more information, see our Statistics Policy.

RESPONSE: A "Data Availability Statement" has been added, detailing where all supporting data and analysis scripts are available.

EDITOR COMMENTS

Reviewing Editor:

Comments for Authors to ensure the paper complies with the Statistics Policy:
Please define N values and report SD (rather than SEM). For data points where n<30, provide the data as supporting information. Provide precise p-values.

RESPONSE: All N values are now clearly defined, SD is reported instead of SEM, data with n < 30 have been provided as Supporting Information, and all p-values are reported precisely in accordance with the Statistics Policy.

Comments to the Author:
The manuscript describes the distribution, maturation, and protein synthesis functions of the ER/SR networks in cardiomyocytes. This manuscript is well-written and the data are clearly presented. The work is recognized to be of broad interest to the field.

RESPONSE: We thank the reviewer for their thoughtful and positive comments. We are glad that the work is considered of broad interest and that the data presentation and manuscript clarity were well received.

There are a few points raised by the reviewers that should be addressed to strengthen the manuscript, including quantification of confocal images, and consideration of whether reduced expression of ER proteins reflects redistribution as cell size increases with maturation.

RESPONSE: We thank the Editor for these helpful comments. We have added quantification of confocal images and included a discussion addressing whether reduced ER protein expression may reflect redistribution as cardiomyocyte size increases during maturation.

Additional comments:

• Please define N values and report SD (rather than SEM).

RESPONSE: All N values are now clearly defined, and standard deviations (SD) are reported instead of SEM.

• Please correct typographical errors in the figures and figure legends (e.g., fluorescence is written 'flourescence')

RESPONSE: Typographical errors in figures and figure legends (e.g., “flourescence”) have been corrected.

• Provide molecular weights for WBs.

RESPONSE: Molecular weights have been added for all Western blots.

• Figure 4 legend describe a panel C that is not found in the provided figure. CHX is not included in the figure legend.

RESPONSE: The Figure 4 legend has been corrected to match the panels shown, and CHX is now properly described.

• Scale bars should be added to the TEM figures in Fig 5A-D

RESPONSE: Scale bars have been added to all TEM images in Figure 5A–D.

• Please include an ethics statement and method of euthanasia.

RESPONSE: An ethics statement, including the method of euthanasia, has been added to the Methods section.

Senior Editor:

Comments to the Author:
I concur with the Reviewing Editor. The manuscript is potentially interesting but would require a major revision to address the concerns raised in the review.

RESPONSE: We thank the Senior Editor for their consideration. We have addressed all concerns raised by the Reviewing Editor, including clarifications, additional quantifications, and revisions throughout the manuscript, in this major revision.

Point-By-Point Responses to Reviewers

Referee #1:

Presented manuscript interrogates molecular differentiation and structural organization of the ER/SR during cardiac muscle development using biochemistry and imaging techniques. The authors demonstrate show that sarcoplasmic reticulum in adult cardiac myocytes possess some classical function of the ER. Namely, it binds active ribosomes and serves as a site of translation for some proteins such as RyR2. Please find my comments/suggestions below:

1) Line : 85 "Since most Ca2+ handling proteins reside in either the lumen of the SR or in the SR membrane [9,12], it is reasonable to posit that contractile Ca2+ handling proteins in cardiac myocytes are synthesized on ribosomes associated with the SR". Definitely not all of them according to PMID: 32353354 and PMID: 25640161. I suggest toning down this statement. Also, what is contractile Ca2+ handling proteins? Did you mean contractile and Ca2+ handling proteins

We thank the reviewer for this critique and for the clarification on terminology. We have corrected the text to read "Ca2+ handling proteins".

Regarding the site of synthesis, we acknowledge that established models (such as those in PMID: 32353354 and 25640161) emphasize the role of the nuclear envelope and juxtanuclear rough ER as primary sites for the biogenesis and sorting of SR-resident proteins like SERCA2a and phospholamban. However, our study specifically focuses on the structural and molecular maturation of these compartments in the adult ventricular myocyte.

Our data demonstrates that as the ER diminishes postnatally, the SR becomes the principal membrane network throughout the cell, and critically, we find that ribosomes and mRNA localize in close proximity to the SR in these adult cells. While we agree that "not all" proteins are synthesized at the SR, our findings suggest that the adult SR evolves to serve as a direct site of protein synthesis and homeostasis. We have revised the statement to be more nuanced, acknowledging the importance of juxtanuclear synthesis while highlighting the distributed synthesis network we observed in the adult state.

Revised Line 96:

" In striated muscle, the majority of Ca2+ handling proteins are localized within the SR lumen or membrane [9,12]. This has led to the hypothesis that the SR may serve as a site for the localized synthesis of these proteins, similar to the co-translational translocation of ER-luminal and transmembrane proteins observed in the ER of non-muscle cells. However, whether the SR in the mature cardiac myocyte functions as a direct site for protein synthesis remains a subject of ongoing investigation."

New Section Added to the Discussion Line 448:

"It is important to note that established models of cardiac protein biogenesis emphasize a centralized synthesis route for SR-resident proteins, such as SERCA2a, phospholamban (PLB), and junction, originating in the juxtanuclear rough ER and the nuclear envelope (PMID: 32353354 and 25640161). These studies highlight a sophisticated trafficking network, including the NEST (Nuclear Envelope/SR Transit) pathway, that utilizes microtubules to transport newly synthesized proteins from the nuclear axis to the cell periphery."

2) Is there any particular reason that different anti-RyR2 antibodies were used for STED and immunochemistry?

We appreciate the opportunity to clarify the use of these antibodies. We utilized the same monoclonal clone (C3-33) for all RyR2 experiments to ensure consistency in epitope binding. The transition from the Abcam-distributed C3-33 (ab2827) to the Thermo Fisher-distributed C3-33 (MA3-916) was due to the discontinuation of the ab2827 catalog number during the study. We performed side-by-side validation of both antibody sources using standard immunofluorescence and confirmed that they yielded identical staining patterns before utilizing the fresh stock for the high-resolution requirements of STED imaging.

Added to STED Methods Line 191:

"RyR2 was localized using a mouse monoclonal antibody (clone C3-33). For initial immunoblotting and immunocytochemistry, clone C3-33 was obtained from Abcam (ab2827); following its discontinuation, the same clone was sourced from Thermo Fisher Scientific (MA3-916) for STED imaging. The specificity and labeling patterns of both preparations were confirmed to be identical."

3) Line 206. Strictly speaking SERCA is not a channel.

We thank the reviewer for this correction. We agree that referring to SERCA2 as a channel is technically inaccurate. We have revised the text to use the more inclusive and accurate term "Ca2+ handling proteins" to encompass both the RyR2 channel and the SERCA2 pump.

Revised Line 206 (Now Line 250):

"Conversely, adult cardiac myocytes exhibit increased relative expression of Ca^{2+} handling proteins (Figure 1B)."

4) Fig. 1.B. Could you please provide MWs for the westerns?

We have updated Figure 1B to include the molecular weight markers (in kDa) for all representative immunoblots.

5) Quantification for imaging studies is missing which is a major concern

We have now included spatial intensity profiles (line-scan analysis) for the representative adult and neonatal myocytes shown in Figures 3 and 5. This analysis quantifies the spatial overlap of the fluorescent signals within the represented cell, demonstrating the precise peaks of ribosome localization relative to the SR and ER membranes.

While these high-resolution images (N = 1 represented) serve to illustrate the spatial organization, the biochemical population statistics ($N \geq 3$ independent biological replicates) provided in Figure 1B confirm the overall developmental shift in protein expression.

6) Does SR/ER functional overlap changes in anyway in cardiac disease?

The reviewer raises an important point regarding the clinical implications of ER/SR spatial organization. While our study focuses on the molecular differentiation during normal postnatal development, existing research suggests that this specialized organization is highly sensitive to pathological stress. In chronic conditions such as heart failure and hypertrophy, myocytes often undergo a 'de-differentiation' characterized by T-tubule disorganization and a redistribution of the SR protein network.

Furthermore, work from our group and others has shown that cardiac stress, such as ischemia and hypertrophy, specifically activates the ATF6 branch of the ER stress response. This activation serves a

protective role by inducing chaperones to manage protein misfolding. However, chronic activation likely leads to an expansion of ER-like quality control machinery into regions typically reserved for specialized SR function. We have added a section to our Discussion addressing how the specialized protein synthesis network we identified may be a target for remodeling in the diseased heart, citing these mechanisms of transcriptional induction and proteostasis.

Added to Discussion Line 477:

"The specialized structural and functional segregation of the ER and SR in the healthy adult heart appears to be highly susceptible to remodeling during pathology. In chronic conditions such as hypertrophy or heart failure, the maintenance of this distinct, distributed synthesis network may be compromised as the cell prioritizes global proteostasis over specialized function. We and others have previously demonstrated that ischemia and pressure overload specifically activate the ATF6 branch of the ER stress response[53–58]. While ATF6 serves a critical protective role by transcriptionally inducing chaperones to prevent protein misfolding[59], its activation also drives metabolic and structural shifts, such as the induction of the mTORC1 activator Rheb to regulate cardiac hypertrophy[56]. This shift suggests that under pathological stress, the myocyte may revert to a more 'ER-dominant' phenotype, reminiscent of early postnatal stages, where the specialized protein synthesis and Ca^{2+}-handling subdomains of the SR are eclipsed by a globalized quality control and growth program. Understanding the balance between the mature, distributed SR network and the stress-induced ER program is critical for identifying how proteostasis failure contributes to the transition from compensated hypertrophy to heart failure."

Referee #2:

Sandmann et al. perform imaging and molecular studies in neonatal and adult rodent cardiomyocytes to assess the abundance and localization of classical secretory pathway and excitation-contraction coupling machinery and uncover distinct localization of protein synthesis machinery at the more classically-defined perinuclear endoplasmic reticulum as well as at the sarcoplasmic reticulum that is specialized for calcium cycling that suggest distinct but overlapping functions of the ER and SR in protein synthesis and translation. These studies support a model whereby protein biosynthesis, processing, and translation occur both at the classical perinuclear ER and the SR that form a contiguous membrane network for protein biosynthesis. These findings are important for the field and likely to be of broad interest to the readership of the J of Physiol. Some concerns with the manuscript and data interpretation are outlined below.

1. The concept that localized translation occurs in cardiomyocytes at the longitudinal SR , although perhaps beyond the scope of the current study and supported by the presence of actively translating ribosomes at the SR by multiple methods reported here,

a) would require imaging of translation of mRNAs encoding resident SR proteins.
b) Similarly, the concept the classical protein secretion occurs preferentially at the perinuclear ER compared to the SR is not fully supported by the current studies.

a) We thank the reviewer for this constructive feedback. We agree that visualizing the translation of specific mRNAs encoding SR-resident proteins (e.g., using SUnSET or Ribopuromycylation assays combined with FISH) would provide definitive proof of localized synthesis. While we agree that such experiments are beyond the scope of the current structural and molecular characterization, we believe our existing data provide a strong foundation for this conclusion.

Specifically, we have shown: (1) the physical association of active, phosphorylated ribosomes (p-RPS6) with the peripheral SR using super-resolution STED microscopy; (2) the biochemical enrichment of ribosomes in SR-heavy adult myocytes; and (3) the localized presence of mRNA in close proximity to SR markers. These findings, when combined with emerging evidence of specialized mRNA targeting in cardiomyocytes (e.g., PMID: 32353354, cited in our manuscript), strongly suggest that the SR serves as a direct site for the translation of resident proteins. We have revised the Discussion to acknowledge this as a likely mechanism while noting that future studies utilizing site-specific translation assays will be necessary to definitively map the 'translatome' of the SR.

<u>We have revised the manuscript to address this concern as follows:</u>

Revised Results, Line 345:

“In neonatal cardiac myocytes, puromycin-labeled nascent polypeptides were distributed throughout the cell, with the highest signal intensity concentrated in the perinuclear region (Figure 4A). In contrast, adult cardiac myocytes displayed a distinct striated distribution of puromycin-labeled nascent polypeptides throughout the cell periphery (Figure 4B). This pattern closely mirrors the striated organization of the peripheral SR network observed in our RyR2 and SERCA2 immunofluorescence (Figure 1D), suggesting that the SR becomes a major site of active translation as the cell matures. The specificity of the puromycin signal was confirmed by cycloheximide co-treatment, which abolished the labeling in both developmental stages.”

New Addition to Discussion, Line 459:

“The localization of mRNA to the SR likely involves specific cis-acting elements within the mRNA transcripts that facilitate transport via the microtubule network, analogous to the 'zipcode' targeting mechanisms observed in neurons53. Given the high metabolic turnover and the massive size of adult myocytes, such localized

translation at the SR would minimize the energetic cost of long-distance protein trafficking and ensure the rapid replacement of Ca2+-handling machinery at the cell periphery."

b) We appreciate the reviewer's caution regarding the spatial limits of the secretory pathway. Our suggestion that secretion occurs *preferentially* at the perinuclear ER is based on the significantly higher density of classic secretory machinery (e.g., KDEL staining) and the predominant localization of the Golgi apparatus in the perinuclear region of adult myocytes.

However, we acknowledge the reviewer's point that secretory elements are also present, albeit at lower densities, in the cell periphery. We have revised the manuscript to clarify that while the initiation and bulk of the classical secretory pathway appear to be concentrated in the perinuclear ER, we cannot rule out the participation of the peripheral SR network in specialized or secondary secretory functions. We have toned down this claim and now describe the ER as a 'primary hub' rather than an exclusive site for secretion.

<u>We have revised the manuscript to address this concern as follows:</u>

Revised Abstract, Line 56:

"Immunocytochemistry further indicated that the ER and SR perform overlapping yet distinct specialized functions, with excitation–contraction coupling localized to the SR, and initiation of secretion appearing to concentrate within the ER."

Revised Results, Line 301:

"Together, our data indicate the presence of a perinuclear ER and a peripheral SR network in adult differentiated cardiac myocytes. While the highest density of classical secretory machinery is localized to the perinuclear ER, suggesting it acts as a primary hub, the presence of these elements in the cell periphery suggests that the SR network may also participate in specialized protein processing."

New sentence Results, Line 337:

"While poly(A)-mRNA FISH provides a global visualization of the transcriptome rather than identifying specific SR-resident transcripts, the close spatial association of total mRNA and active ribosomes with the SR suggests a high capacity for localized translation within this peripheral network."

Figure 2 Legend:

"The classical secretory machinery and Golgi apparatus are predominantly localized to the perinuclear endoplasmic reticulum in neonatal and adult cardiac myocytes."

Figure 4C Legend:

"(C) Schematic diagram of the sarco/endoplasmic reticulum network in a cardiac myocyte, depicting the <u>proposed</u> relationships between the SR [A] the peri-nuclear ER [B] and nuclear envelope [C]. The localization of secreted and membrane protein synthesis to the nuclear envelope, peri-nuclear ER, and the SR, depicted as a contiguous membranous system, is shaded purple."

Revised Discussion, Line 424:

“Our imaging data suggest that classical secretory pathway activity is concentrated primarily within the perinuclear ER, where the density of quality control chaperones and Golgi markers is highest. While this supports a model where the initiation of secretion is a predominantly perinuclear event, our observations also revealed the presence of secretory machinery in the cell periphery, albeit at a lower relative abundance. This aligns with findings by Bogdanov et al. and suggests that while the ER exhibits greater specialization for protein secretion, the SR may maintain a capacity for specialized or localized secretory functions. Therefore, we propose that the ER serves as the primary hub for the classical secretory pathway, while acknowledging the potential for distributed activity across the contiguous ER-SR network.”

Revised Discussion, Line 470:

“Our findings indicate that the SR in adult cardiac myocytes represents a previously unrecognized site of localized translation. While definitive evidence of localized synthesis would require the direct visualization of SR-specific mRNA translation, our observation of active ribosomes and mRNA localized to the SR network, particularly in the mature, SR-dominant state, provides compelling evidence for this model. This is consistent with the paradigm of polarized cells, such as neurons, which utilize localized translation to maintain protein homeostasis at distal functional sites.”

2. The authors demonstrate reduced expression of classical resident ER proteins anmd ribosomal markers in adult cardiomyocytes compared to neonatal cardiomyocytes concomitant with upregulation of RYR2 and conclude mature adult cardiomycoytes downregtulate secretory pathway machinery. Is it possible that the content of ribosomse/secretory pathway machinery etc. actually remain constant as CMs mature but become distributed in an orderly manner throughout the much larger cell as the cytoarchitecture is remodeled and the cytoplasmic space is filled with well-aligned myofilaments and mitochondria, thereby allowing for coordinated placement of translational machinery at ER and SR membranes throughout the adult cardiomyocyte to allow for efficient protein synthesis? Related, it would be interesting to see if relative myofilament protein and mitochonrdrial protein content are increased in adult vs. neonatal cardiomyoyctes. This may be challenging for myosin isoforms that switch but blotting for sarcomeric actin would be pretty straight forward. Also related, the immunofluoresence signal for translational machinery at the periphery/SR may be more diffuse as the translational machinery is distributed amongst a larger area whereas it is heavily concentrated in a smaller area in the perinuclear ER that although is significant may be somewhat misleading as to the relative abundance of ribosomes/translational machinery in the two regions.

We thank the reviewer for this nuanced perspective on the maturation of the cardiac myocyte protein synthesis network. The reviewer suggests that the apparent 'downregulation' of ER/ribosomal markers in adult cells may reflect a strategic redistribution throughout the significantly larger volume of the mature myocyte, rather than an absolute loss of machinery.

We agree that the dramatic expansion of myofibrillar and mitochondrial mass in the adult myocyte, which we have addressed in the revised Discussion, effectively 'dilutes' the relative concentration of the secretory and translational apparatus.

Revised Discussion, Line 411:

“Indeed, analysis of global transcriptomic trends during mammalian heart development indicates that while sarcomeric and mitochondrial genes become the most abundant transcripts in the adult, the absolute number of translational components does not scale linearly with total cell volume (Brawand et al., 2011). This suggests that the apparent downregulation of ER and ribosomal markers we observed in adult myocytes likely reflects a "dilution" effect within the total proteome as the cytoplasmic space is filled with well-aligned myofilaments and mitochondria. Consistent with this, we observed an expansion of the SR from neonatal to adult myocytes. In adults, the SR extends throughout much of the cellular periphery, while the ER remains confined to a smaller perinuclear region. This transition represents an orderly redistribution of the translational machinery, moving

from a concentrated perinuclear hub in the relatively small neonatal cell to a distributed network in the adult, facilitating coordinated protein synthesis across the vastly expanded sarcoplasmic volume.”

Discussion, Line 428:

“It is important to note that the intense immunofluorescence signal for translational machinery in the perinuclear region may be influenced by the high density of the organelle in that small volume. In contrast, the machinery in the cell periphery is distributed across a significantly larger surface area of the striated SR, which may result in a more diffuse signal despite a significant total abundance of peripheral ribosomes.”

3. Major title captions for figure legends for Figures 1 and 2 do not accurately summarize the presented data and/or findings and should be rewritten.

We thank the reviewer for this suggestion. We agree that the original titles were too broad and did not fully capture the developmental and structural nuances of the data. We have revised the titles for Figures 1 and 2, focusing on the developmental expansion of the SR and the specific spatial localization of the secretory machinery.

Figure 1 now emphasizes the postnatal maturation and the relative shift in ER/SR markers:

“Postnatal maturation of cardiac myocytes is characterized by the expansion of the sarcoplasmic reticulum and a relative decrease in endoplasmic reticulum markers”

Figure 2 now accurately describes the localization of the secretory machinery rather than making a functional claim about the site of secretion itself:

“The classical secretory machinery and Golgi apparatus are predominantly localized to the perinuclear endoplasmic reticulum in neonatal and adult cardiac myocytes.”

4. The claim that distinct localization patterns of SERCA2 and RyR2 in neonatal cardiomyocytes likely represent forming T-tubules is unlikely to be the case as T-tubules do not begin forming until well over a week in rodent cardiomyocytes.

We thank the reviewer for this important clarification regarding the timeline of T-tubule development. We agree that at postnatal days 1–2, structural T-tubules have not yet formed in rodent myocytes. Our original phrasing was intended to describe the nascent junctions or the "pre-tubular" organization of RyR2 at the cell surface, which precedes the eventual invagination of the sarcolemma. We have revised the Results section to use more precise terminology, referring to these structures as nascent junctions with the sarcolemma rather than "forming T-tubules," to align with the established developmental timeline.

Revised Results, Line 268:

“The distinct localization patterns of SERCA2 and RyR2 likely reflect the developing membrane network and mark sites of nascent junctions with the sarcolemma. While structural T-tubules do not fully develop in rodents until later in the postnatal period, these peripheral RyR2 clusters represent the early spatial organization required for the eventual establishment of excitation-contraction coupling machinery.”

5. Fig legend 4C describes graphical schematic that is not present in the current figure (and may be worth including?)

We have added this diagram to Figure 4C. Thank you.

6. TEM images in Figs 5A-D need scale bars.

Revised Figure 5A-D now includes scale bars.

7. Introduction discussion of junctional SR should include description of its close proximity to the T-tubules.

This is now revised on line 91.

8.Typographical/grammatical errors: line 89, 111, 116, 139.

We apologize for these errors. We have corrected the typographical and grammatical mistakes on the specified lines and have performed a thorough proofreading of the entire manuscript to ensure clarity and technical accuracy.

Dear Dr Doroudgar,

Re: JP-RP-2026-288658R1 "Development of a protein synthesis network at the sarco/endoplasmic reticulum in adult cardiac myocytes" by Christoph Sandmann, Soeren Brandenburg, Dennis Uhlenkamp, Bianca Meyer, Clara Sandmann, Nicole Herzog, Julia Groß, Charlotta Funaya, Hugo Katus, Stephan E Lehnart, Christopher C Glembotski, Mirko Völkers, and Shirin Doroudgar

Thank you for submitting your manuscript to The Journal of Physiology. It has been assessed by a Reviewing Editor and by 2 expert referees and we are pleased to tell you that it is acceptable for publication following satisfactory revision.

The referee reports are copied at the end of this email.

Please address all the points raised and incorporate all requested revisions or explain in your Response to Referees why a change has not been made. We hope you will find the comments helpful and that you will be able to return your revised manuscript within 4 weeks. If you require longer than this, please contact journal staff: jp@physoc.org.

Your revised manuscript should be submitted online using the link in your Author Tasks Link Not Available. This link is accessible via your account as Corresponding Author; it is not available to your co-authors. If this presents a problem, please contact journal staff (jp@physoc.org). Image files from the previous version are retained on the system. Please ensure you replace or remove any files that are being revised.

If you do not wish to submit a revised version of your manuscript, you must inform our journal staff (jp@physoc.org) or reply to this email to request withdrawal. Please note that a manuscript must be formally withdrawn from the peer review process at one journal before it may be submitted to another journal.

TRANSPARENT PEER REVIEW POLICY: To improve the transparency of its peer review process The Journal of Physiology publishes online, as supporting information, the peer review history of all articles accepted for publication. Readers will have access to decision letters, including Editors' comments and referee reports, for each version of the manuscript, as well as any author responses to peer review comments. Referees can decide whether or not they wish to be named on the peer review history document.

ABSTRACT FIGURES: Authors are expected to use The Journal's premium BioRender account to create/redraw their Abstract Figures. Information on how to access this account is here: https://physoc.onlinelibrary.wiley.com/journal/14697793/biorender-access.

This will enable Authors to create and download high-resolution figures. If authors have used the free BioRender service, they can use the instructions provided in the link above to download a high-resolution version suitable for publication.

The link provided should only be used for the purposes of this submission. Authors will be charged for figures created on this account if they are not related to this manuscript submission.

LANGUAGE EDITING AND SUPPORT FOR PUBLICATION: If you would like help with English language editing, or other article preparation support, Wiley Editing Services offers expert help, including English Language Editing, as well as translation, manuscript formatting, and figure formatting at www.wileyauthors.com/eeo/preparation. You can also find resources for Preparing Your Article for general guidance about writing and preparing your manuscript at www.wileyauthors.com/eeo/prepresources.

RIGOUR AND REPRODUCIBILITY: We recommend authors consult The Journal's Rigour and Reproducibility requirements to ensure that their manuscript is suitable for publication: https://physoc.onlinelibrary.wiley.com/pb-assets/hub-assets/physoc/documents/TJP-Rigour-and-Reproducibility-Requirements.pdf

REVISION CHECKLIST:

Check that your Methods section conforms to journal policy: https://jp.msubmit.net/cgi-bin/main.plex?form_type=display_requirements#methods

Check that data presented conforms to the statistics policy: https://jp.msubmit.net/cgi-bin/main.plex?form_type=display_requirements#statistics

Upload a full Response to Referees file. To create your 'Response to Referees' copy all the reports, including any comments from the Senior and Reviewing Editors, into a Microsoft Word, or similar, file and respond to each point, using font or background colour to distinguish comments and responses and upload as the required file type.

Please upload two versions of your manuscript text: one with all relevant changes highlighted and one clean version with no changes tracked. The manuscript file should include all tables and figure legends, but each figure/graph should be uploaded as separate, high-resolution files. The journal is now integrated with Wiley's Image Checking service. For further details,

see: https://www.wiley.com/en-us/network/publishing/research-publishing/trending-stories/upholding-image-integrity-wileys-image-screening-service

You may also upload:

- 'Potential Cover Art' for consideration as the issue's cover image
- Appropriate Supporting Information (Video, audio or data set: see https://jp.msubmit.net/cgi-bin/main.plex?form_type=display_requirements#supp).

We look forward to receiving your revised submission.

If you have any queries, please reply to this email and we will be pleased to advise.

Yours sincerely,

Bjorn Knollmann
Senior Editor
The Journal of Physiology

EDITOR COMMENTS

Reviewing Editor:

Ethics Concerns:
Please add in a statement to the methods with the ethical approval information.

Comments to the Author:
Thank you for your response and for addressing the reviewers' points.

Please revise the manuscript to include an ethics statement in the methods (animal experiments section) and add in the additional organelle labels for Figure 5A and 5D.

Senior Editor:

Comments to the Author:
I concur with the reviewing editor's assessment. The MS will be acceptable after correcting a few minor issues.

REFEREE COMMENTS

Referee #1:

The manuscript interrogates molecular differentiation and structural organization of the ER/SR during cardiac muscle development using biochemistry and imaging techniques. The authors demonstrate that sarcoplasmic reticulum in adult cardiac myocytes possess some classical function of the ER. Namely, it binds active ribosomes and serves as a site of translation for some proteins such as RyR2.

My previous concerns were addressed. No further questions.

Referee #2:

Sandmann et al. perform imaging and molecular studies in neonatal and adult rodent cardiomyocytes to assess the abundance and localization of classical secretory pathway and excitation-contraction coupling machinery and uncover distinct localization of protein synthesis machinery at the more classically-defined perinuclear endoplasmic reticulum as well as at the sarcoplasmic reticulum that is specialized for calcium cycling that suggest distinct but overlapping functions of the ER and SR in protein synthesis and translation.

These studies support a model whereby protein biosynthesis, processing, and translation occur both at the classical perinuclear ER and the SR that form a contiguous membrane network for protein biosynthesis. These findings are important for the field and likely to be of broad interest to the readership of the J of Physiol.

I recommend electron micrographs in Fig 5A of ribosomes at perinuclear membranes and Fig 5D of ribosomes at "subsarcolemmal region...at the intercalated disc" clearly show and label the nucleus and intercalated disc, respectively. All of my concerns have been addressed in the very well-written revised manuscript.

END OF COMMENTS

Dear Dr. Bjorn Knollmann, Reviewing Editor, and the Reviewers,

The authors appreciate the continued positive evaluation of our manuscript and the helpful suggestions to finalize the paper. We have addressed the remaining minor requests regarding the ethics statement and figure labeling as detailed below.

Responses to Editor's Comments

REQUIRED ITEMS

Ethics Concerns:

- **Comment:** Please add in a statement to the methods with the ethical approval information.
- **RESPONSE:** An explicit ethics statement has been moved from the end of the manuscript to the beginning of the Methods section. This statement includes specific institutional ethical approval information and oversight committee details, ensuring full compliance with the Journal's requirements and enhanced visibility.

Figure Labels & EM Methods:

- **Comment:** Please revise the manuscript to include... additional organelle labels for Figure 5A and 5D.
- **RESPONSE:** We have updated Figure 5A and Figure 5D with the requested structural labels and expanded the Methods section to include a comprehensive description of our electron microscopy procedures. Specifically, we have labeled the nucleus (N) in Figure 5A to provide spatial orientation for the perinuclear region. In Figure 5D, we added labels for the intercalated disc region (ID) and terminal sarcolemma (TS) to clearly define the subsarcolemmal boundaries and terminal pole. Furthermore, the Methods section now includes a detailed protocol for our EM sample preparation and imaging parameters to ensure full reproducibility of these structural findings.

Responses to Referee #1

- **Comment:** The manuscript interrogates molecular differentiation and structural organization of the ER/SR... My previous concerns were addressed. No further questions.
- **RESPONSE:** We thank the reviewer for their careful evaluation throughout this process and for their positive assessment of our revised manuscript.

Responses to Referee #2

- **Comment:** I recommend electron micrographs in Fig 5A of ribosomes at perinuclear membranes and Fig 5D of ribosomes at "subsarcolemmal region...at the intercalated disc" clearly show and label the nucleus and intercalated disc, respectively. All of my concerns have been addressed in the very well-written revised manuscript.
- **RESPONSE:** We appreciate the reviewer's kind words regarding the clarity of our revision. To address the final recommendation, we have updated the electron micrographs and the associated methods as follows:
    - Figure 5A: The nucleus (N) is now clearly labeled to show the spatial relationship between the perinuclear ER and the nuclear envelope.
    - Figure 5D: The intercalated disc region (ID) and terminal sarcolemma (TS) are now labeled. As these images represent isolated cardiomyocytes, these labels identify the terminal pole and the region where the intercalated disc was previously situated, providing the necessary structural context for the localized ribosomal clusters observed.
    - Detailed EM Methods: We have expanded the Methods section to include a robust description of the EM fixation, embedding, and imaging protocols used to capture these high-resolution details.

In summary, we have implemented all requested ethical and labeling corrections. We believe these final adjustments have further sharpened the clarity and reproducibility of our structural findings, and we look forward to the publication of our work in the *Journal of Physiology*.

Dear Dr Doroudgar,

Re: JP-RP-2026-288658R2 "Development of a protein synthesis network at the sarco/endoplasmic reticulum in adult cardiac myocytes" by Christoph Sandmann, Soeren Brandenburg, Dennis Uhlenkamp, Bianca Meyer, Clara Sandmann, Nicole Herzog, Julia Groß, Charlotta Funaya, Hugo Katus, Stephan E Lehnart, Christopher C Glembotski, Mirko Völkers, and Shirin Doroudgar

We are pleased to tell you that your paper has been accepted for publication in The Journal of Physiology.

*** As per your recent correspondence with the Editorial Office, would you like to update the legend to Figure 1 before the article goes to production? Please email a new legend to: jp@physoc.org ***

The last Word (or similar) version of the manuscript provided will be used by the Production Editor to prepare your proof. When this is ready you will receive an email containing a link to Wiley's Online Proofing System. The proof should be thoroughly checked and corrected as promptly as possible.

Authors should note that it is too late at this point to offer corrections prior to proofreading. Major corrections at proof stage, such as changes to figures, will be referred to the Editors for approval before they can be incorporated. Only minor changes, such as to style and consistency, should be made at proof stage. Changes that need to be made after proof stage will usually require a formal correction notice.

All queries at proof stage should be sent to: TJP@wiley.com.

Following copyediting and typesetting the accepted version will be published online.

Yours sincerely,

Bjorn Knollmann
Senior Editor
The Journal of Physiology

IMPORTANT POINTS TO NOTE FOLLOWING ACCEPTANCE OF YOUR PAPER:

• IMPORTANT NOTICE ABOUT OPEN ACCESS: To assist authors whose funding agencies mandate immediate public access to published research findings, The Journal of Physiology allows authors to pay an Open Access (OA) fee to have their papers made freely available immediately on publication.

The Corresponding Author will receive an email from Wiley with details on how to register or log in to Wiley Authors where you will be able to place an order.

• You can check if your funder or institution has a Wiley Open Access Account here:
https://authors.wiley.com/author-resources/Journal-Authors/open-access/author-compliance-tool.html

• TRANSPARENT PEER REVIEW POLICY: To improve the transparency of its peer review process, The Journal of Physiology publishes online as supporting information the peer review history of all articles accepted for publication. Readers will have access to decision letters, including Editors' comments and referee reports, for each version of the manuscript as well as any author responses to peer review comments. Referees can decide whether or not they wish to be named on the peer review history document.

• You can help your research get the attention it deserves! Check out Wiley's free Promotion Guide for best-practice recommendations for promoting your work at: www.wileyauthors.com/eeo/guide. You can learn more about Wiley Editing Services which offers professional video, design, and writing services to create shareable video abstracts, infographics, conference posters, lay summaries, and research news stories for your research at: www.wileyauthors.com/eeo/promotion.

• If you would like to receive our 'Research Roundup', a monthly newsletter highlighting the cutting-edge research published in The Physiological Society's family of journals (The Journal of Physiology, Experimental Physiology, Physiological Reports, The Journal of Nutritional Physiology and The Journal of Precision Medicine: Health and Disease), please click this link, fill in your name and email address and select 'Research Roundup':
https://www.physoc.org/journals-and-media/membernews

EDITOR COMMENTS

Reviewing Editor:

Thank you for addressing all of the review comments.

Senior Editor:

The manuscript is now acceptable for publication. Thank you for your excellent contribution to the Journal!
